# Selective C−C Coupling by Spatially Confined Dimeric Metal Centers

**DOI:** 10.1016/j.isci.2020.101051

**Published:** 2020-04-12

**Authors:** Yanyan Zhao, Si Zhou, Jijun Zhao

**Affiliations:** 1 Key Laboratory of Materials Modification by Laser, Ion and Electron Beams (Dalian University of Technology), Ministry of Education, Dalian 116024, China

**Keywords:** Catalysis, Atomic Electronic Structure, Energy Sustainability, Numerical Method in Materials Science

## Abstract

Direct conversion of carbon dioxide (CO_2_) to high-energy fuels and high-value chemicals is a fascinating sustainable strategy. For most of the current electrocatalysts for CO_2_ reduction, however, multi-carbon products are inhibited by large overpotentials and low selectivity. Herein, we exploit dispersed 3d transition metal dimers as spatially confined dual reaction centers for selective reduction of CO_2_ to liquid fuels. Various nitrogenated holey carbon monolayers are shown to be promising templates to stabilize these metal dimers and dictate their electronic structures, allowing precise control of the catalytic activity and product selectivity. By comprehensive first-principles calculations, we screen the suitable transition metal dimers that universally have high activity for ethanol (C_2_H_5_OH). Furthermore, remarkable selectivity for C_2_H_5_OH against other C_1_ and C_2_ products is found for Fe_2_ dimer anchored on C_2_N monolayer. The role of electronic coupling between the metal dimer and the carbon substrates is thoroughly elucidated.

## Highlights

• Dual metal centers provide an exclusive pathway for CO_2_ reduction to C_2_ products
• Activity and selectivity are modulatable by the metal-support interaction
• Fe_2_ dimer anchored on C_2_N leads to remarkable selectivity for ethanol

## Graphical Abstract

**Figure:**
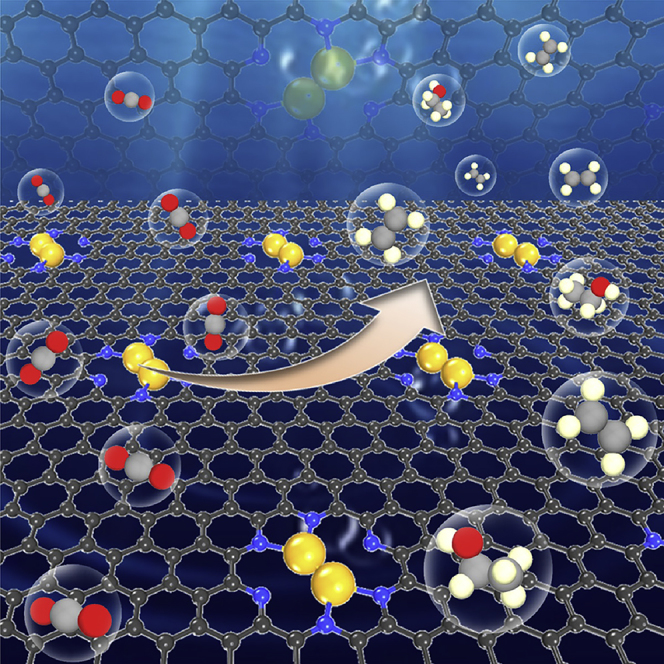

## Introduction

Production of liquid fuels by catalytic convertion of CO_2_, the main greenhouse gas and meanwhile an abundant carbon feedstock, has been regarded as an appealing strategy to solve both energy and environmental crises, albeit facing great challenges (Birdja et al., 2019, Jia et al., 2019, Amal et al., 2017). Copper-based materials have been widely adopted as catalysts for electro-reduction of CO_2_ to multi-carbon (C_2_ or C_2+_) products (Zheng et al., 2019). Although fairly good activity can be achieved by modification or morphology engineering of copper, such as sculpturing it into nanoparticles or nanocubes, doping or alloying, and making oxide-derived copper, the selectivity and efficiency of most copper-based electrocatalysts remain unsatisfactory for commercialization of the CO_2_ conversion technique to high-energy fuels and high-value chemicals (Gao et al., 2019, Kim et al., 2017, Wang et al., 2018a, Zhou et al., 2018).

Recenlty, transition metal atoms dispersed on nitrogen-doped porous carbon nanomaterials emerge as a promising category of electrocatalysts for CO_2_ reduction, which have maximum atomic efficiency, high electrical conductivity and good durability, and can be facilely synthesized in the laboratory (Bayatsarmadi et al., 2017, Chen et al., 2019a, Cheng et al., 2018, Wang et al., 2019a). The transition metal atoms are usually anchored in the pores of the carbon matrix and coordinated with the nitrogen atoms, exhibiting unique electronic states and acting as isolated reaction centers for CO_2_ reduction. Remarkable activity and selectivity toward carbon monoxide (CO) has been observed for various dispersed transition metal atoms (Fe, Co, Ni, Mn, and Cu) on N-doped graphene, carbon nanosheets or nanospheres, with selectivity up to 97% and Faradaic efficiency above 80% (Jiang et al., 2018a, Ren and Zhao, 2020, Wang et al., 2019b, Yang et al., 2018, Zhang et al., 2018). First-principle calculations show that the activity highly depends on the type of metal atoms, which provide different binding strengths with the reaction intermediates (Ju et al., 2017). The single metal sites also have an advantage of suppressing the competing hydrogen evolution reaction (HER), due to the unique adsorption configuration of H^∗^ species compared with those on the transition metal surfaces (Bagger et al., 2017).

Furthermore, homonuclear and heteronuclear dimers of transition metal immobilized in carbon-based nanostructures, such as Fe_2_ and Fe-Co on nitrogenated graphitic carbon materials, Fe-Ni on N-doped graphene, and Pt-Ru on g-C_3_N_4_, have been synthesized in the laboratory (Wang et al., 2017, Wang et al., 2018b, Ye et al., 2019, Zhou et al., 2019). This opens up the windows for a broader range of chemical processes that require dual reaction centers either with enhanced activity or carrying different functionalities simultaneously. For instance, Ren et al. fabricated diatomic Fe-Ni sites embedded in nitrogenated carbon (Ren et al., 2019). By taking advantage of the strong binding capability of Fe with CO_2_ molecule and the weak adsorption of CO on Ni, they achieved impressively high selectivity of 99% for CO and Faradaic efficiency above 90% over a wide potential range from −0.5 to −0.9 V, reaching 98% at −0.7 V versus reversible hydrogen electrode (RHE). On the theoretical side, a Cu_2_ dimer supported on the C_2_N monolayer was predicted to have high selectivity for methane (CH_4_), whereas dimerization of two CO species leading to the formation of ethene (C_2_H_4_) is possible with an energy cost of 0.76 eV (Zhao et al., 2018). Heteronuclear dimers such as V-Mo on 2D C_2_N and Cu-B on g-C_3_N_4_ have been shown to effectively reduce CO_2_ to ethanol (C_2_H_5_OH) and C_2_H_4_, owing to the synergistic interaction and asymmetric coupling between two reaction centers yielding favorable binding strength for the formation of C_2_ intermediates (Li et al., 2019, He et al., 2020).

Two adjacent metal atoms that are spatially confined in a hole of N-doped carbon materials as unique active sites not only enable the simultaneous fixation of two CO_2_ molecules but also sterically limit the reaction pathways that may be beneficial for C−C coupling toward C_2_ or C_2+_ products. Moreover, various combinations of metal dimers and carbon substrates give high degrees of freedom for modulating the catalytic performance. However, the atomistic mechanism and composition recipe of such heterogeneous catalysts remain largely unknown, which impede their rational design and experimental synthesis for practical uses.

Here we exploit 3*d* transition metal dimers immobilized on various nitrogenated holey carbon sheets for selective reduction of CO_2_ to C_2_ products. By systematic first-principle calculations, the detailed C−C coupling mechanism on the spatially confined dual metal centers has been elucidated for the first time. The suitable transition metal elements and carbon substrates that lead to high activity and selectivity for C_2_H_5_OH and C_2_H_4_ are screened, and the underlying electronic structure-activity relationship is unveiled. These theoretical explorations illuminate important clues for precisely engineering the dispersed metal catalysts on porous carbon nanomaterials for direct conversion of greenhouse gas to multi-carbon hydrocarbons and oxygenates.

## Results and Discussion

**Figure 1:**
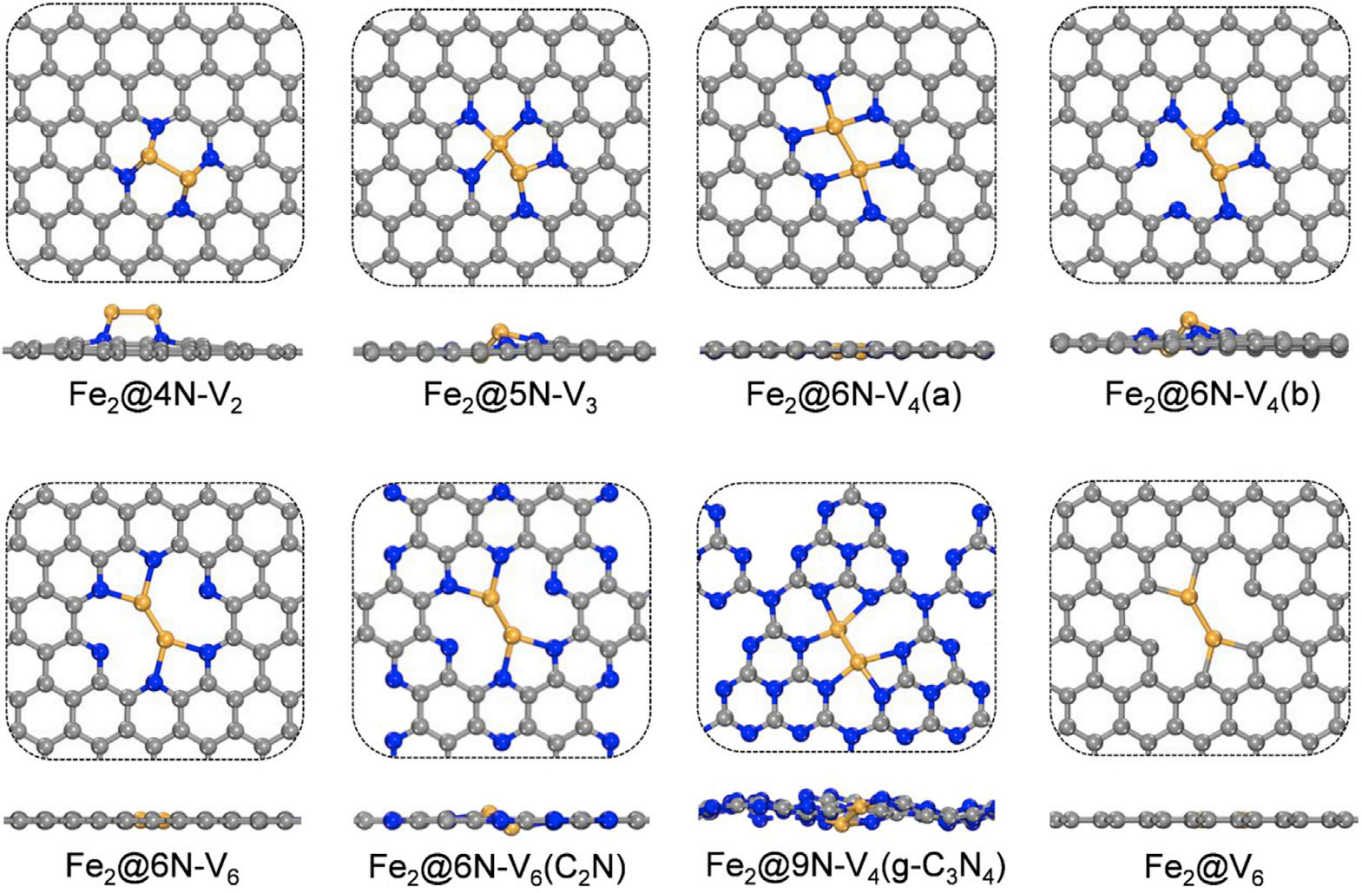
Atomic Structures of a Fe_2_ Dimer Anchored on Various Nitrogenated Holey Carbon Monolayers (Top Panel: Top View; Bottom Panel: Side View) The C, N, and Fe atoms are shown in gray, blue, and orange colors, respectively.

In the laboratory, N-doped graphitic carbon materials with controllable doping contents (up to 16.7% of N content) and atomic geometries can be achieved via either direct synthesis or posttreatment (Xue et al., 2012, Xu et al., 2018, Qu et al., 2010). Here we focused on pyridine N dopants in graphene, which are the main doping species at high N contents and are usually associated with the vacancies or pores of the carbon basal plane (Sheng et al., 2011, Sarau et al., 2017). As displayed in Figure 1, we considered a series of N-doped holey graphene monolayers, comprising C vacancies of various sizes (denoted as V_*n*_, *n* = 2, 3, 4, 6) with the edges coordinated with different numbers of N atoms (denoted as *m*N, *m* = 4, 5, 6). Specifically, 4N-V_2_, 5N-V_3_, and 6N-V_4_ systems can be viewed as four, five, and six N atoms decorating the edges of di-vacancy, tri-vacancy, and tetra-vacancy in graphene, respectively, all of which have been commonly observed in experiment (He et al., 2014, Lin et al., 2015, Wang et al., 2018c). Note that the V_6_ pore in graphene is a favorable defect according to transmission electron microscopy experiment (Robertson et al., 2015), and our previous calculation showed that N-doped V_6_ (namely 6N-V_6_) has extraordinary thermodynamic stability (Luo et al., 2013). We further created a number of randomly N-doped graphene lattices, which shows that the 6N-V_6_ configuration would emerge as N doping content reaches 10% (see Figure S1 for details). Besides the N-doped graphitic sheets, we also considered the synthetic carbon nitride monolayers, including g-C_3_N_4_ and C_2_N (Zhao et al., 2014, Mahmood et al., 2015). All these porous N-coordinated carbon sheets have formation energies (defined by Equation S1 in Supplemental Information) in the range of 0.16–0.21 eV/Å, whereas the N-free V_6_ is higher in energy by over 0.46 eV/Å than the others (Table 1). These nitrogenated 2D holey carbon materials are ideal templates to stabilize and disperse metal atoms or small clusters. Indeed, isolated Fe_2_, Fe-Ni, and Fe-Co dimers embedded in 6N-V_4_(a), as well as Fe_2_ and Pt-Ru dimers anchored on g-C_3_N_4_ have already been realized in experiment (Ye et al., 2019, Wang et al., 2017, Zhou et al., 2019, Ren et al., 2019, Tian et al., 2018).

**Table 1:** Structural and Energetic Properties of Supported Fe_2_ Dimer

Substrate	*E*_form_ (eV/Å)	*E*_b_ (eV)	*d* (Å)		CT (*e*)	Δ*E*_CO2∗_ (eV)
			Fe−Fe	N−Fe		
4N-V_2_	0.17	−5.01	2.09	1.98	0.71	−1.02
5N-V_3_	0.19	−7.33	1.91	1.87	0.97	−1.15
6N-V_4_(a)	0.20	−9.47	2.21	1.94	0.96	−0.11
6N-V_4_(b)	0.21	−7.48	2.14	1.98	0.81	−1.15
6N-V_6_	0.16	−6.02	1.96	2.00	0.76	−1.12
C_2_N	–	−5.80	2.01	1.97	0.74	−0.64
g-C_3_N_4_	–	−5.09	1.98	1.99	0.72	−1.58
V_6_	0.67	−12.03	2.21	1.94	0.87	−0.30

Formation energy (*E*_form_) of various nitrogenated 2D holey carbon materials, binding energy (*E*_b_) of a Fe_2_ dimer on the carbon sheet, bond length (*d*) of Fe−Fe and N−Fe/C−Fe bonds, Mulliken charge transfer (CT) from Fe_2_ to the carbon sheet, and adsorption energy of a CO_2_ molecule (Δ*E*_CO2∗_) on the supported Fe_2_ dimer.

To evaluate the capability of various supported metal dimers for CO_2_ reduction toward C_2_ products, we first explored the atomic structures, electronic and adsorption properties of dimeric 3*d* transition metal clusters on the 6N-V_6_ monolayer (as will be shown later, this substrate gives metal dimers the highest activity for CO_2_ reduction). As presented in Figures 1 and S2, all metal dimers are embedded in the hole of the graphitic sheet, except that Sc_2_ with a larger atomic size induces a noticeable buckling of 0.94 Å in the out-of-plane direction. Four N−metal bonds are formed with bond length of 1.95–2.09 Å, and the metal−metal bond length ranges from 1.96 Å to 2.79 Å (Table 2). The binding energy (defined by Equation S2 in Supplemental Information) between the metal dimer and the graphitic sheet is −4.29 to −10.28 eV, excluding the possibility of dissociation or aggregation of the metal dimer. The thermal stability of these carbon-substrate-anchored metal dimers was further assessed by *ab initio* molecular dynamics (AIMD) simulations, which manifest that they can sustain at least 800 K for 10 ps with small vertical displacement of metal atoms (<0.2 Å) (see Figure S3 for details), suggesting superior thermal stability for practical uses.

**Table 2:** Structural and Energetic Properties of Various Supported 3*d* Transition Metal Dimers

Metal Dimer	*E*_b_ (eV)	*d* (Å)		Δ*E* (eV)		*ε*_d_ (eV)
		M−M	N−M	CO_2_	2CO_2_	
Sc_2_	−10.28	2.79	2.09	−3.40	−3.71	1.16
Ti_2_	−8.50	2.17	1.99	−2.85	−3.31	0.62
V_2_	−8.80	2.14	1.96	−2.26	–	0.41
Cr_2_	−4.99	2.16	1.97	−1.44	−0.76	0.07
Mn_2_	−6.52	2.04	2.01	−1.05	−0.48	−0.42
Fe_2_	−6.02	1.96	2.00	−1.12	−0.50	−1.00
Co_2_	−5.71	2.10	1.95	−1.20	–	−1.09
Ni_2_	−5.93	2.17	2.00	−0.82	–	−1.12
Cu_2_	−4.29	2.35	1.96	−0.35	–	−2.08

Binding energy (*E*_b_) of various 3*d* transition metal dimers anchored on the 6N-V_6_ monolayer, bond lengths (*d*) of metal dimer (M−M) and N−metal (N−M), adsorption energy of single and dual CO_2_ molecules on the supported metal dimers (Δ*E*), and the *d* band center (*ε*_d_) of the supported metal dimers (Hammer and Nørskov, 2000).

**Figure 2:**
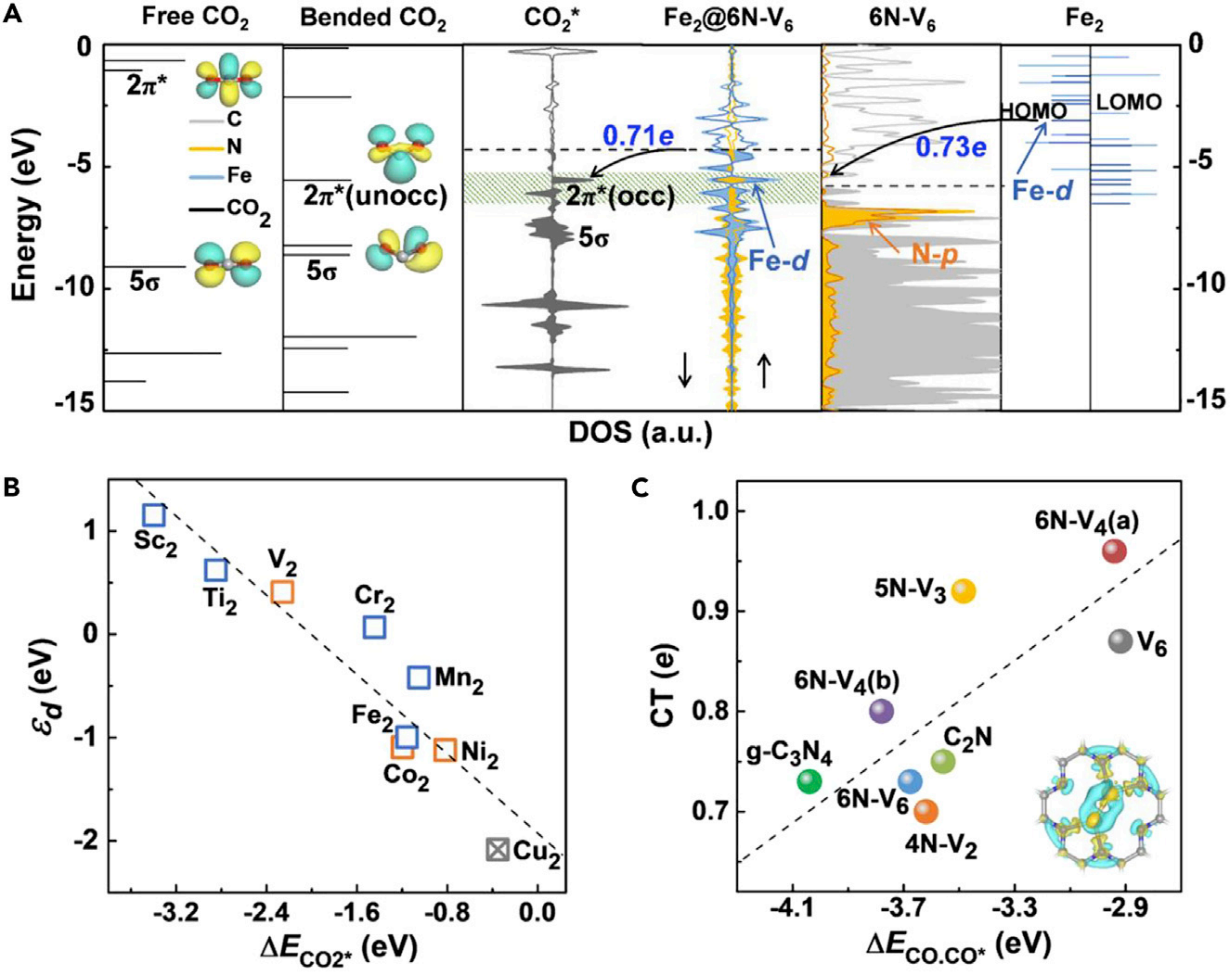
Electronic Structure-Activity Relationship (A) From left to right: molecular orbital levels or local density of states (DOS) of a free and a bended (with C−O−C angle of 130°) CO_2_ molecule in vacuum, an adsorbed CO_2_ molecule on Fe_2_@6N-V_6_, an individual 6N-V_6_ monolayer, and a Fe_2_ dimer. The insets display the HOMO and LUMO charge densities of CO_2_. The energy is relative to the vacuum. The dashed line shows the Fermi level, with the occupied states shadowed. The hybridization region between *d* orbital of Fe atoms and 2π∗ state of CO_2_ is shadowed in green. The dark blue and orange colors represent the *d* orbital of Fe atoms and p orbital of N atoms, respectively. (B) The *d* band center (*ε*_d_) of various supported 3*d* transition metal dimers as a function of the adsorption energy of single CO_2_ molecule. The blue/orange/gray symbols denote that two/one/none CO_2_ molecule can be chemisorbed on the metal dimer. The dashed line is a linear fit of the data points. (C) Charge transfer (CT) from the Fe_2_ dimer to various nitrogenated carbon holey monolayer as a function of the adsorption energy of dual CO molecules. The dashed line is a linear fit of the data points. The insert shows the differential charge density of Fe_2_@6N-V_6_. The yellow and cyan colors represent the electron accumulation and depletion regions, respectively, with an isosurface value of 0.005 *e*/Å^3^.

**Figure 3:**
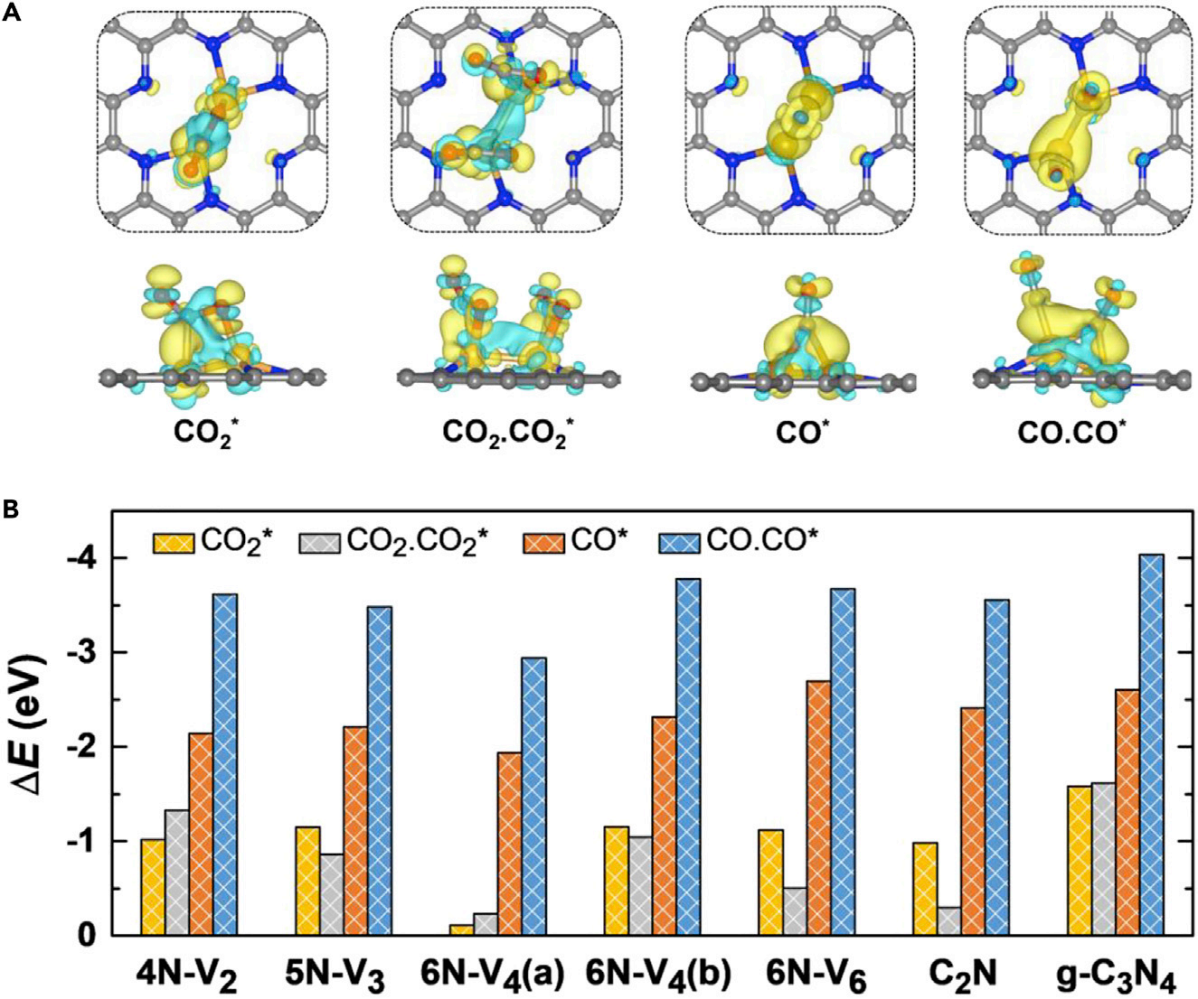
Structures and Energies of Molecular Adsorption (A) From left to right: differential charge densities of single and dual CO_2_ molecules, single and dual CO molecules adsorbed on Fe_2_@6N-V_6_. The yellow and cyan colors represent the electron accumulation and depletion regions, respectively, with an isosurface value of 0.005 *e*/Å^3^. (B) Adsorption energies of single and dual CO_2_ and CO molecules on the Fe_2_ dimer anchored on various nitrogenated holey carbon monolayers. The C, N, O, and Fe atoms are shown in gray, blue, red, and orange colors, respectively.

A CO_2_ molecule can favorably chemisorb on these dispersed metal dimers except Cu_2_. The molecule is bended in the bidentate configuration with O−C−O angle of 124.90–141.96°. The C atom and one of the O atoms of CO_2_ form two bonds with the underlying metal atoms; the C−O bond length is elongated to 1.21–1.36 Å, compared with 1.16 Å for a free CO_2_ molecule. The dynamic process of CO_2_ adsorption was also examined by AIMD simulations at 100 K and 300 K, respectively, both showing that the molecule can quickly chemisorb on the dimeric metal centers within a simulation time of 1 ps (see Videos S1 and S2 for the dynamic movies). The adsorption energy (defined by Equation S3 in Supplemental Information) of CO_2_ ranges from −0.82 eV to −3.40 eV. Overall speaking, stronger binding is provided by the metal element with fewer *d* electrons. The trend of activity can be understood by the electronic density of states (DOS) shown in Figure 2A. Taking Fe_2_@6N-V_6_ as an example, hybridization between the *d* orbitals of Fe_2_ dimer and the *p* orbitals of 6N-V_6_ monolayer substrate is evident, with prominent electronic states close to the Fermi level mainly contributed by the Fe atoms (see Figure S4 for projected DOS). Electron transfer of 0.73 *e* occurs from Fe_2_ to 6N-V_6_ monolayer, which lifts the Fermi level of the hybrid system above the 2π^∗^ state of CO_2_. As a result, Fe_2_@6N-V_6_ can favorably donate about 0.71 electrons to the antibonding orbital of CO_2_, as manifested by the differential charge densities in Figure 3A, which is a general mechanism for activation of reactant molecules on metal active centers (Liu et al., 2018). As depicted in Figure 2B, CO_2_ adsorption energy generally follows a linear relationship with the *d* band center of the supported metal dimers (relative to the Fermi level), as the metal dimer with a higher *d* band center would provide stronger binding with CO_2_ (Hammer and Nørskov, 2000).

**Figure 4:**
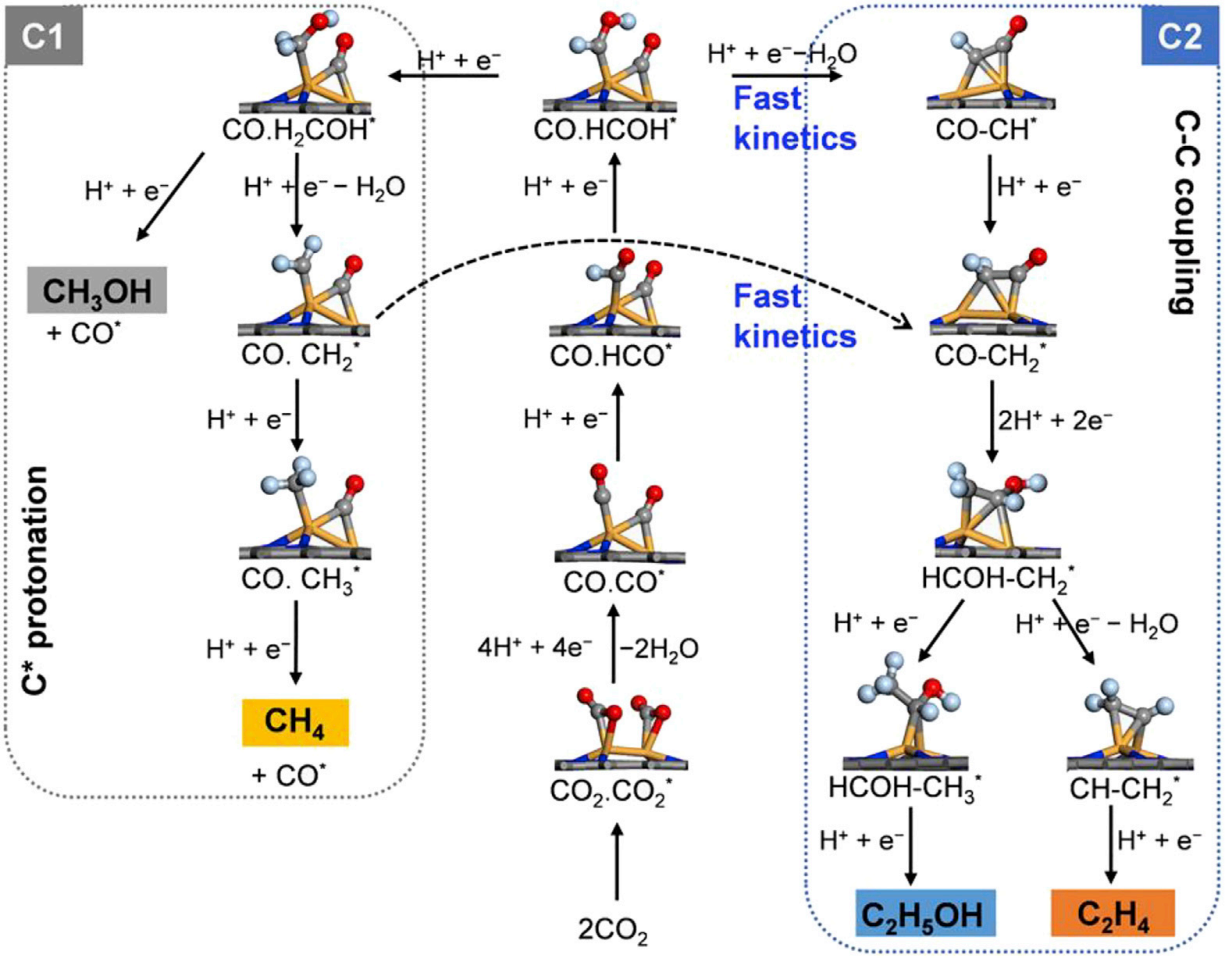
The CO_2_ reduction pathways to various C_1_ and C_2_ products on the supported Fe_2_ dimer The H, C, N, O, and Fe atoms are shown in light blue, gray, blue, red, and orange colors, respectively.

Video S1. The Dynamic Process of CO_2_ Adsorption on Fe_2_@6N-V_6_ at 100 K by AIMD Simulations, Related to Figure 4

Video S2. The Dynamic Process of CO_2_ Adsorption on Fe_2_@6N-V_6_ at 300 K by AIMD Simulations, Related to Figure 4

In addition, we examined the capability of various dispersed 3*d* transition metal dimers for activating two CO_2_ molecules simultaneously, which is a prerequisite for C−C coupling to yield C_2_ products. Several candidate systems including Sc_2_, Ti_2_, Cr_2_, Mn_2_, and Fe_2_ dimers on the 6N-V_6_ monolayer have adsorption energies of −3.71 to −0.48 eV for fixation of two CO_2_ molecules (Figures 3 and S5), whereas the other metal dimers are only able to bind one CO_2_ molecule. Considering that Fe is an earth-abundant element and dispersed Fe atoms and dimers can be readily obtained in the experiment (Ye et al., 2019, Tian et al., 2018), thereafter we explored Fe_2_ dimer on various nitrogenated 2D holey carbon materials as a representative of dual metal centers.

Figure 1 presents the structures of a Fe_2_ dimer immobilized on several 2D carbon substrates. The dimer forms 4–6 bonds with the neighboring N or C atoms, having bond lengths of 1.91–2.21 Å for Fe−Fe and 1.87–2.00 Å for N−Fe (C−Fe) bonds, respectively, and the binding energies are −5.01 to −12.03 eV (Table 1). The Fe_2_ dimer exhibits different buckling height in the out-of-plane direction (0.01–2.06 Å) and meanwhile induces some local vertical distortions on the carbon basal plane (0.09–0.35 Å). The dimer-substrate coupling strength depends on the size of the hole as well as the saturation degree of the edge atoms. For instance, binding strength between Fe_2_ and 4N-V_2_, 5N-V_3_, and 6N-V_4_(a) increases with both N content and hole size. The bonding interaction between Fe_2_ and g-C_3_N_4_ or C_2_N is relatively weak, due to the electronic saturation of these two semiconducting carbon nitride monolayers (as manifested by their large band gaps). In sharp contrast, Fe_2_ is strongly anchored on the nitrogen-free V_6_ defect that has six unsaturated carbon atoms on the hole edge, thereby leading to the largest binding energy of −12.03 eV.

All the supported Fe_2_ dimers are able to chemisorb two CO_2_ molecules with total adsorption energies of −0.23 to −1.62 eV (compared with −0.11 to −1.58 eV for adsorption of single CO_2_ molecule), as revealed by Figure 3B. Our nudged elastic band (NEB) calculations show that adsorption of the second CO_2_ molecule involves kinetic barriers of 0.29–1.04 eV. Both CO_2_ molecules are bended with O−C−O angle of 141.00–152.13° and elongated C−O bond lengths of 1.17–1.29 Å. The C atom in each CO_2_ is bonded to the underlying Fe atom with Fe−C bond length of 1.93–2.12 Å. Furthermore, we investigated the interaction between the dispersed Fe_2_ dimers and the CO molecule, which is an important reaction intermediate in the CO_2_ reduction process. Our calculations indicate strong binding of CO on the anchored Fe_2_ dimers, with adsorption energies of −2.94 to −4.04 eV (−1.94 to −2.70 eV) for two (one) CO molecules. Consequently, desorption of CO from dual metal centers would be rather difficult, which allows further protonation of CO and thus provides the opportunity for successive C−C coupling.

The distinct binding capability of various supported Fe_2_ dimers with gas molecules can be related to the electronic coupling between Fe_2_ and the carbon substrate. As displayed in Figure 2C, the amount of charge transfer from Fe_2_ to the substrate varies from 0.71 *e* to 0.97 *e*. Generally speaking, less electron transfer leads to higher activity of the Fe_2_ dimer for CO_2_ and CO chemisorption, which is consistent with the trend of binding energies between Fe_2_ and the carbon templates discussed before (Table 1). It is the N content, the degree of electronic saturation of the hole edge, and the bond configuration of Fe_2_ in the hole that jointly determine the coupling strength between the metal dimer and the carbon sheets. Therefore, the nitrogenated 2D holey carbon materials with diverse morphologies and controllable N contents can not only stabilize and disperse metal dimers but also dictate the electronic structures and activity of the anchored metal dimers. By choosing proper metal elements and substrates, it is possible to delicately mediate their coupling strength and charge transfer, endowing large degree of freedom to optimize the activity and selectivity of various supported metal dimers for CO_2_ reduction.

Note that graphitic N species are inevitably present in the experimentally synthesized N-doped carbon materials (Lin et al., 2014). To clarify their effect on the activity of the dispersed Fe_2_ dimer, we investigated the CO_2_ adsorption on Fe_2_@6N-V_6_ containing various numbers of graphitic N atoms at different distances from the 6N-V_6_ hole (Figure S6). For all the considered systems, the CO_2_ adsorption energies on the catalysts with and without substitutional N atoms on the graphene lattice differ by less than 0.16 eV, suggesting that existence of the graphitic N species has only minor impact on the catalytic properties of the Fe_2_ dimer supported on pyridine holes of 2D carbon substrates.

**Figure 5:**
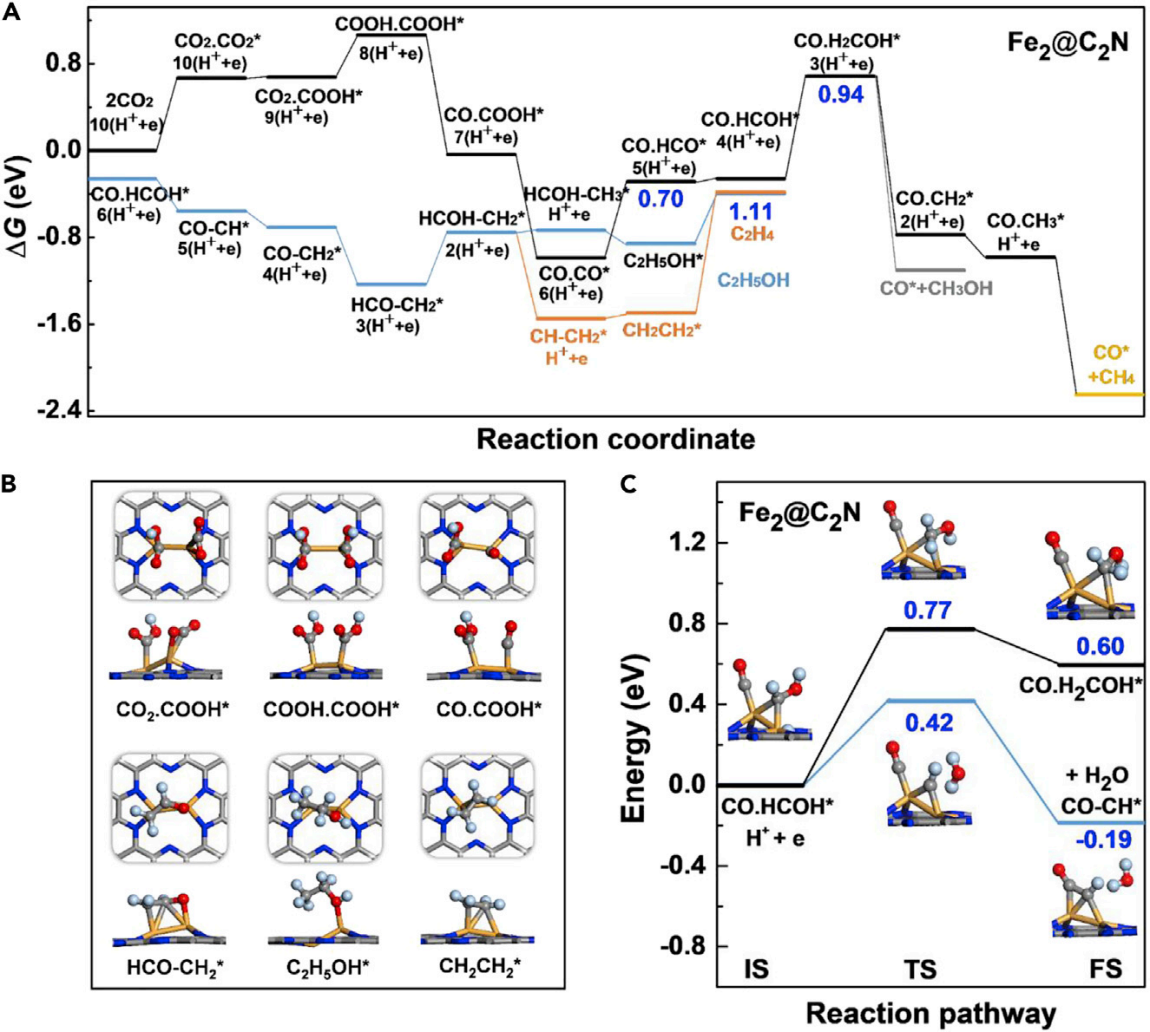
CO_2_ Reduction Pathway (A and B)(A) Free-energy diagram of CO_2_ reduction to various C_1_ and C_2_ products (indicated by different colors) on Fe_2_@C_2_N. The blue numbers, from left to right, give the Gibbs free energy of formation for the rate determining step of C_2_H_5_OH, C_2_H_4_, and CH_3_OH/CH_4_. The local structures of selected reaction intermediates are presented in (B). The H, C, N, O, and Fe atoms are shown in light blue, gray, blue, red, and orange colors, respectively. (C) Competing reactions of CO.HCOH^∗^ to form C_1_ and C_2_ intermediates on Fe_2_@C_2_N. The insets display the structures of initial state (IS), transition state (TS), and final state (FS). The blue numbers give the kinetic barriers (middle) and heat of reaction (right).

Figure 4 shows the most efficient pathways for CO_2_ reduction toward possible C_1_ and C_2_ products on the Fe_2_ dimer immobilized on various nitrogenated carbon sheets, and the corresponding free-energy diagrams of various model systems calculated by the computational hydrogen electrode (CHE) model (Peterson et al., 2010) are given by Figures 5, S7, and S8. We used point (.) to represent the co-adsorption of two carbon intermediates on the catalyst and strigula (−) to indicate the coupling between two carbon intermediates. The maximum Gibbs free energy of formation Δ*G* among all the reaction steps defines the rate-determining step (RDS) and is thus denoted as Δ*G*_RDS_. Overall speaking, formation of C_2_ products first requires the activation of dual CO_2_ molecules on the catalyst. By going through the carboxyl (COOH^∗^) pathway, two CO^∗^ intermediates can be generated; then protonation of CO^∗^ leads to C_1_ products such as methanol (CH_3_OH) and CH_4_. Alternatively, it paves a way to the coupling between two neighboring carbon intermediates, which is energetically favorable and kinetically easy, and finally yields C_2_ products (C_2_H_5_OH and C_2_H_4_). A similar path for C−C coupling was also found for the other metal dimers anchored on the nitrogenated carbon sheet, as revealed by Figure S9 for Ni_2_@6N-V_6_ as an example.

Specifically, formation of two CO^∗^ species on most of the considered Fe_2_ dimers is uphill in the free-energy profile, involving energy steps of 0.24–0.87 eV. Then, reduction of CO^∗^ gives rise to HCO^∗^ species, which is lower in energy by up to 1.13 eV than the other possible intermediates such as COH^∗^ (Figure S10). The CO^∗^ → HCO^∗^ conversion is endothermic with Δ*G* = 0.49–1.01 eV. Further protonation of HCO^∗^ leads to HCOH^∗^ and then produces a CH^∗^ species by release of a H_2_O molecule. The C−C coupling reaction is most likely to occur between a CH^∗^ (or CH_2_^∗^) species and the neighboring CO^∗^. Our NEB calculations suggest that the CO−CH^∗^ coupling is exothermic and barrierless on all the considered Fe_2_ dimers, except for Fe_2_@C_2_N and Fe_2_@C_3_N_4_ that involve a small kinetic barrier of about 0.22 eV (Table S1). According to previous theoretical studies (Goodpaster et al., 2016, Jiang et al., 2018b), Cu(211) and (100), as the typical active surfaces for CO_2_ reduction, favor dimerization of CO^∗^ or CO−HCO^∗^ coupling involving Δ*G* = −0.17–0.48 eV. For the present Fe_2_ dimers on nitrogenated carbon sheets, however, CO−CO^∗^ or CO−HCO^∗^ coupling has higher Δ*G* than the values of CO−CH^∗^ by 0.84–2.42 eV and thus is unlikely to occur.

Following the C−C coupling, successive reduction of CO−CH^∗^ leads to CO−CH_2_^∗^, HCO−CH_2_^∗^, HCOH−CH_2_^∗^, HCOH−CH_3_^∗^, and finally yields C_2_H_5_OH. Alternatively, reduction of HCOH−CH_2_^∗^ can give rise to CH−CH_2_^∗^ with release of a H_2_O molecule, and further protonation of CH−CH_2_^∗^ eventually produces C_2_H_4_. These elementary reactions involve relatively small steps of 0.15–0.73 eV in free-energy profile and thus would take place readily from the thermodynamic point of view. At the last step, desorption of C_2_H_5_OH^∗^ and CH_2_CH_2_^∗^ is endothermic by 0.11–0.59 eV and 0.23–1.69 eV, respectively. For most of the considered Fe_2_ dimers, the rate-determining step for C_2_H_5_OH production is the CO^∗^ → HCO^∗^ conversion. The release of C_2_H_4_ mainly suffers from the strong binding of CH_2_CH_2_^∗^ on the catalyst, which can be overcome by the reaction heat of the corresponding reduction step (0.61–2.07 eV) (Chen et al., 2019b), as well as by adopting some strategies such as the pulse electrolysis mode to accelerate desorption of the final products (Yano et al., 2007, Qiao et al., 2014).

On the other hand, formation of C_1_ products is also possible on the dispersed Fe_2_ dimers. As discussed earlier, HCOH^∗^ can be reduced to CH^∗^, followed by the CO−CH^∗^ coupling. Alternatively, HCOH^∗^ may be protonated to H_2_COH^∗^. Then, reduction of H_2_COH^∗^ yields CH_3_OH or produces CH_2_^∗^ with release of a H_2_O molecule followed by the generation of CH_3_^∗^ and CH_4_. For Fe_2_@4N-V_2_, Fe_2_@6N-V_6_, and Fe_2_@g-C_3_N_4_, the CO^∗^ → HCO^∗^ conversion is the rate determining step for both C_1_ products. For Fe_2_@6N-V_4_(b) and Fe_2_@5N-V_3_, formation of CH_3_OH from H_2_COH^∗^ protonation requires Δ*G*_RDS_ = 1.45 and 0.98 eV, respectively. In particular, Fe_2_@C_2_N encounters Δ*G*_RDS_ = 0.94 eV and a kinetic barrier of 0.77 eV during the reaction of HCOH^∗^ → H_2_COH^∗^ for both C_1_ products, whereas the competing step of CO−HCOH^∗^ → CO−CH^∗^ + H_2_O has much reduced Δ*G* = −0.30 eV and a lower kinetic barrier of 0.42 eV (Figures 5A and 5C). This would lead to high selectivity for C_2_ products on Fe_2_@C_2_N.

**Figure 6:**
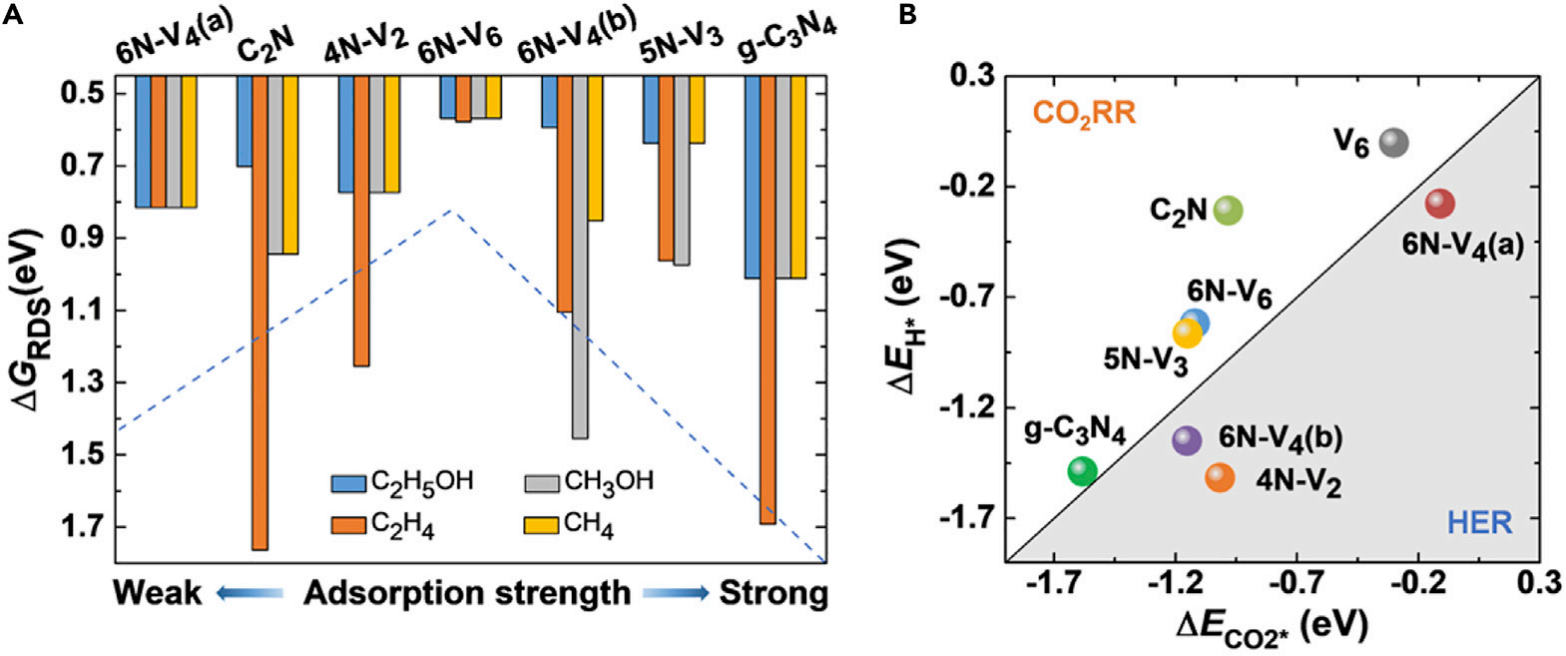
Catalytic Performance for CO_2_ Reduction (A) Gibbs free energy of formation for the rate determining step (Δ*G*_RDS_) for various C_1_ and C_2_ products from CO_2_ reduction, and (B) competition between adsorption of a CO_2_ molecule and an H^∗^ species on the Fe_2_ dimer anchored on various nitrogenated holey carbon monolayers.

Figure 6A plots Δ*G*_RDS_ values for various C_1_ and C_2_ products from CO_2_ reduction on the anchored Fe_2_ dimers. Among the four products, C_2_H_5_OH exhibits the lowest Δ*G*_RDS_ = 0.57–1.01 eV, and the highest activity is achieved by Fe_2_@6N-V_6_ owing to its moderate adsorption strength with the reaction intermediates (indicated by the dashed blue line in Figure 6A). Formation of C_2_H_4_ is less favorable with Δ*G*_RDS_ = 0.58–1.76 eV due to the strong binding of CH_2_CH_2_^∗^ on the Fe_2_ dimers. Fe_2_@6N-V_4_(a), Fe_2_@4N-V_2_, Fe_2_@6N-V_6_, and Fe_2_@g-C_3_N_4_ exhibit similar selectivity for C_2_H_5_OH, CH_3_OH, and CH_4_, whereas Fe_2_@5N-V_3_ favors both C_2_H_5_OH and CH_4_ products. Remarkable selectivity for C_2_H_5_OH is obtained for Fe_2_@C_2_N and Fe_2_@6N-V_4_(b) with Δ*G*_RDS_ = 0.70 and 0.59 eV, respectively, notably lower than Δ*G*_RDS_ values for the other products (above 0.94 and 0.85 eV, respectively). Hence, these supported Fe_2_ dimers have competitive activity but distinct selectivity with regard to the conventional Cu-based catalysts. It is known that Cu crystals mainly produce CO under low electrode potentials, whereas CH_4_ and C_2_H_4_ are the main products at sufficiently high electrode potentials (about −1.0 V versus RHE in experiment) (Dai et al., 2017, Mistry et al., 2016). Previous calculations revealed that Cu(211) surface encounters Δ*G*_RDS_ = 0.74 eV for CH_4_ and C_2_H_4_, whereas formation of CO is much more favorable with Δ*G*_RDS_ = 0.41 eV due to the relatively weak adsorption of CO on the Cu surface (adsorption energy Δ*E* = −1.01 eV) (Peterson et al., 2010). Differently, release of CO is prohibited on the present Fe_2_ dimers that have strong adsorption energy of Δ*E* = −2.94 to −4.04 eV with CO molecule.

As the electroreduction of CO_2_ usually take place in the neutral aqueous condition, we further explored the solvent effect on the catalytic behavior of supported Fe_2_ dimer. As a representative, we considered an explicit solvent model of Fe_2_@C_2_N. Our calculations show that hydrogen bonds are formed between water molecules and some adsorbed reaction intermediates (such as CO_2_.CO_2_^∗^ and CO−CH^∗^), which slightly stabilize those species on the catalyst in aqueous environment, consistent with the previous theoretical report (Zhao and Liu, 2020). The variations of CO_2_ adsorption energy and Gibbs free energy of formation for elementary steps are below 0.29 eV, and the kinetic barriers of rate determining steps for various products increase by less than 0.35 eV, with regard to the model in vacuum (see Table 3 and Figure S11 for details). The predicted selectivity is consistent between the model in vacuum and in water. Therefore, free-energy calculations on electrocatalysis of CO_2_ reduction using a model of catalyst in vacuum can generally predict reliable results on the trend of activity and product selectivity (De Luna et al., 2018, Zhuang et al., 2018, Li et al., 2018). Besides solvent effect, the surface charge on catalysts during the electrochemical reaction may modify the electronic states and impact the catalytic properties according to a previous theoretical report (Kim et al., 2018). Future studies with sophisticated model theory are necessary to comprehensively evaluate the catalytic performance of the proposed transition metal dimers on the nitrogenated carbon substrates.

**Table 3:** Key Reaction Steps in Vacuum and Aqueous Condition

	Step	CO_2_→ CO_2_^∗^	CO_2_^∗^ + CO_2_→ CO_2_.CO_2_^∗^	CO.CH^∗^ → CO−CH^∗^	CO.HCOH^∗^ + H^+^ + e^−^→	
					CO.H_2_COH^∗^	CO−CH^∗^ + H_2_O
Vacuum	Δ*H* *E*_a_	−0.64 0	0.68 0.70	0.11 0.23	0.60 0.77	−0.19 0.42
Aqueous	Δ*H* *E*_a_	−0.65 0	0.51 0.74	−0.03 0.58	0.31 0.93	−0.18 0.66

The reaction energy (Δ*H*) and kinetic barriers (*E*_a_) for activating first and second CO_2_ molecule, CO−CH^∗^ coupling, and protonation of CO.HCOH^∗^ to CO.H_2_COH^∗^ species or CO−CH^∗^ with H_2_O molecule in vacuum and in the aqueous condition, respectively, given in the unit of eV.

The unique geometry and favorable adsorption properties of the Fe_2_ dimers immobilized on carbon substrates bring about inimitable advantages for their catalytic behavior. First, CO, as an inevitable and even dominant product of CO_2_ reduction on many metal catalysts, severely limits the formation of higher-energy-density products (Zhu et al., 2014, Sarfraz et al., 2016, Peng et al., 2018), but it would be largely suppressed on the anchored Fe_2_ dimers. Second, the adjacent dual metal centers and their strong binding with CO pave an efficient pathway for C−C coupling reaction; in contrast, C−C coupling only occurs on metal surfaces with homogenously distributed reaction sites when the coverage of CO is sufficiently high (Morales-Guio et al., 2018, Huang et al., 2017). Third, the difficult desorption of C_2_H_4_ from the Fe_2_ dimers may result in superior selectivity for C_2_H_5_OH, which is a clean liquid fuel with high heating value. For most of the Cu based catalysts, however, the yield of C_2_H_5_OH is quite low compared with C_2_H_4_ (Liang et al., 2018).

At last, we assess the activity of these supported Fe_2_ dimers for HER, which is a competing reaction against CO_2_ reduction and highly affects the efficiency of CO_2_ conversion (Zhu et al., 2016, Cui et al., 2017). Figure 6B plots the competition between adsorption of H^∗^ species and CO_2_ molecule on the Fe_2_ dimers. The H^∗^ adsorption energy ranges from −1.52 eV to −0.28 eV. For Fe_2_@5N-V_3_, Fe_2_@6N-V_6_, Fe_2_@C_2_N, and Fe_2_@C_3_N_4_, the adsorption strength of H^∗^ species is notably weaker than that of CO_2_ molecule by 0.09–0.67 eV, implying that CO_2_ reduction would prevail over HER on these catalysts with either high activity or superior selectivity. For Fe_2_@4N-V_2_, Fe_2_@6N-V_4_(a), and Fe_2_@6N-V_4_(b), the H^∗^ adsorption strength is stronger than that of CO_2_, which may suppress the CO_2_ reduction. Combining the information in Figures 6A and 6B, we conclude that four of our considered systems are eligible for catalysis of CO_2_ reduction with high activity for C−C coupling toward C_2_ products. Among them, Fe_2_@C_2_N has remarkable selectivity for ethanol; Fe_2_@5N-V_3_ favors the formation of both ethanol and methane; Fe_2_ on g-C_3_N_4_ and 6N-V_6_ have lower selectivity and may generate both C_1_ and C_2_ products. Therefore, dimeric transition metal clusters immobilized on the nitrogenated holey carbon substrates form a category of efficient electrocatalysts for reduction of CO_2_ to high-value hydrocarbons and alcohols, with desired selectivity achievable by choosing proper substrate.

### Conclusion

In summary, we exploited dispersed 3*d* transition metal dimers for CO_2_ reduction to selectively produce liquid fuels. Comprehensive first-principles calculations show that nitrogenated holey carbon materials not only serve as templates to stabilize small metal clusters but also dictate their electronic structures. Specifically, controlling the metal-substrate coupling strength allows effective modulation of both activity and product selectivity. As a consequence, the spatially confined dual reaction centers within the carbon matrix exhibit the following advantageous catalytic behavior: (1) simultaneous fixation of two CO_2_ molecules, (2) prohibition of CO desorption, and (3) exclusive pathway for C−C coupling with high activity. The selectivity is tunable by choosing proper substrate materials. In particular, a Fe_2_ dimer embedded in the C_2_N monolayer exhibits remarkable selectivity for C_2_H_5_OH against the other C_1_ and C_2_ products as well as HER. These theoretical findings provide vital knowledge of the design rules of subnano metal clusters for converting greenhouse gas to high-energy fuels and high-value chemicals and meanwhile call for more experimental and theoretical efforts to advance the technologies for precise synthesis of atomically dispersed catalysts with well-controlled composition and structures.

### Limitations of the Study

This study systematically exploited 3*d* transition metal dimers anchored on nitrogenated holey carbon monolayers for selective reduction of CO_2_ to liquid fuels and screened suitable metal elements and carbon templates with high selectivity for ethanol. However, experimental realization of such superior subnano catalysts relies on the preparation of metal clusters with specific size supported on some given substrates, which may be challenging and requires the development of advanced synthesis methods.

## Methods

All methods can be found in the accompanying Transparent Methods supplemental file.

## Supplemental Information

Document S1. Transparent Methods, Figures S1–S11, and Tables S1–S4

## Footnotes

Supplemental Information can be found online at https://doi.org/10.1016/j.isci.2020.101051.
