# Supplementary material for: Selective C−C Coupling by Spatially Confined Dimeric Metal Centers
Source: iScience. 2020 Apr 12;23(5):101051. doi: 10.1016/j.isci.2020.101051 (PMC7183208; doi:10.1016/j.isci.2020.101051)
Supplement: Document S1. Transparent Methods, Figures S1–S11, and Tables S1–S4 [file mmc1.pdf]

iScience, Volume *23*

Supplemental Information

Selective C−C Coupling by Spatially Confined Dimeric Metal Centers

Yanyan Zhao, Si Zhou, and Jijun Zhao

Supplemental Information

Transparent Methods

Computational Methods

Our calculations were performed using the Vienna *ab initio* simulation package (VASP) based on spin-polarized density functional theory (DFT) (Kresse and Furthmüller, 1996), with the projector augmented wave (PAW) method for ion-electron interaction (Kresse and Joubert, 1999), and the generalized gradient approximation (GGA) parameterized by Perdew, Burke, and Ernzerhof (PBE) for the exchange and correction functional (Perdew et al., 1996). We used the planewave basis set with the energy cutoff of 500 eV. The Grimme's semiempirical DFT-D3 scheme of dispersion correction was adopted for a reliable description of the interactions between catalysts and reaction species (Grimme, 2010). To incorporate a metal dimer, we considered defective graphene sheets comprising holes, whose edge atoms are partially substituted by N atoms (shown in Figure 1). The supercell consists of 7 × 7 graphene unit cells with a lattice constant of 17.22 Å for the lateral directions, and a vacuum space of 20 Å for the perpendicular direction. We also considered two kinds of monolayer carbon nitrides with stoichiometry of C_2N and g-C_3N_4, using 2 × 2 unit cells with supercell dimensions of 16.64 Å and 14.26 Å, respectively. One metal dimer was placed in the hole of each model supercell structure. The Brillouin zones of all the supercells were sampled by the 2 × 2 × 1 Monkhorst-Pack ***k*** grids. The geometry optimization was carried out for both ionic and electronic degrees of freedom, with the convergence criteria of 10^{-4} eV and 0.02 eV/Å for energy and force, respectively. On-site charges were evaluated by the Mulliken population analysis (Mulliken, 1955) implemented in the CASTEP (Clark et al., 2005), using the planewave basis with energy cutoff of 1000 eV, the norm-conserving pseudopotentials, and the PBE functional. The climbing image nudged elastic band method was employed to search for the transition states of elementary steps and calculate the kinetic barriers (Henkelman et al., 2000).

The thermodynamic stability of a defective carbon substrate was characterized by

the formation energy per unit length defined as

$$E_{\text{form}} = (E_{\text{NxCy}} - x/2\ E_{\text{N2}} - yE_{\text{C}})\ /\ L \tag{S1}$$

where E_{NxCy} is the energy of the carbon substrate comprising x numbers of N atoms and y numbers of C atoms per supercell; E_{N2} and E_{C} are the energies of a N_2 gas molecule and a C atom in perfect graphene, respectively; L is the total edge length of a hole in the carbon substrate. The binding strength between the metal dimer and the carbon substrate was evaluated as

$$E_{\text{b}} = E_{\text{M2@NxCy}} - E_{\text{M2}} - E_{\text{NxCy}} \tag{S2}$$

where E_{M2} and $E_{\text{M2@NxCy}}$ are the energies of the metal dimer (M_2) in vacuum and on the carbon substrate, respectively. The thermal stability of these carbon substrate anchored metal dimers was further assessed by *ab initio* molecular dynamics (AIMD) simulations.

To characterize the interaction between reaction molecules (CO_2 and CO) and catalyst, we defined the adsorption energy as

$$\Delta E\ = E_{\text{total}} - E_{\text{M2@NxCy}} - E_{\text{mol}} \tag{S3}$$

where E_{total} is the energy of the molecule adsorbed on the catalyst; E_{mol} is the energy of the molecule in gas phase. The Gibbs free energy of formation (ΔG) for each reaction step was calculated by including the zero-point energy and entropy (Table S3 and S4). The maximum ΔG among all the reaction steps defines the rate-determine step (RDS) and is thus denoted as ΔG_{RDS}. Then, the free energy diagrams of CO_2 reduction were obtained based on the computational hydrogen electrode model (Peterson et al., 2010).

Random simulation of N-doped carbon monolayer

To obtain the lattice structures of various N-doped carbon monolayers, we used a home-made program to generate random structures of graphene lattice with different N doping concentrations. The essential idea of the program is to produce a graphene lattice of a given size and add certain number of N dopants into it. Specifically, a lattice C atom is randomly selected to be substituted by N atom or deleted and replacing its adjacent sites with N atoms until the total number of N atoms reaches the target N content. There are three types of N atom: graphitic N, pyridinic N, and pyrrolic N. Experimentally, pyridinic N dopants in graphene are the major doping species at high

N contents (Sheng et al., 2011; Sarau et al., 2017). Thus, we mainly considered N contents from 3.6% to 15.6% (with pyridinic N and graphitic N of 3.3% ~ 15.3% and 0.3%, respectively). For each N content, we created several random lattice structures of N-doped graphene, as shown in Figure S1. In generally, the types and size of the holes increase as the nitrogen concentration increases. Particularly, 4N-V_2, 5N-V_3 and 6N-V_4 exist as the N content lower than 7.5%, and 6N-V_6 would arise as the N doping content reaches 10%.

Dynamic movie of the MD simulation on CO_2 adsorption process

As a respective, we have performed calculations to explore the dynamic process of CO_2 adsorption on Fe_2@6N-V_6 at 100 K (Supplemental Video S1) and 300 K (Supplemental Video S2) by AIMD simulations, respectively. The results show that the molecule can quickly chemisorb on the dimeric metal centers within a simulation time of 1 ps. These two movies are provided as independent files for download. Related to Figure 4.

Table S1. Adsorption energies of single and dual CO molecules (ΔE_{CO*}, $\Delta E_{CO.CO*}$), kinetic barriers of CO−CH and CO−CH_2 coupling, and the Gibbs free energy of formation for the rate-determine step (ΔG_{RDS}) of CH_3OH, CH_4, C_2H_4 and C_2H_5OH for the Fe_2 dimer anchored on various carbon substrates. Related to Figure 4.

Substrate	ΔE_{CO*} (eV)	$\Delta E_{CO.CO*}$ (eV)	Kinetic barrier (eV)		ΔG_{RDS} (eV)			
			CO−CH	CO−CH_2	CH_3OH	CH_4	C_2H_4	C_2H_5OH
4N-V_2	−2.14	−3.62	0	0.53	0.77	0.77	1.25	0.77
5N-V_3	−2.21	−3.48	0	0.49	0.98	0.64	0.96	0.64
6N-V_4(a)	−1.94	−2.94	0	0.62	0.82	0.82	0.82	0.82
6N-V_4(b)	−2.31	−3.78	0	0.48	1.45	0.85	1.11	0.59
6N-V_6	−2.70	−3.68	0	0	0.57	0.57	0.58	0.57
C_2N	−2.41	−3.56	0.23	0.38	0.94	0.94	1.76	0.70
g-C_3N_4	−2.61	−4.04	0.22	0.19	1.01	1.01	1.69	1.01

Table S2. Adsorption energies of second CO_2 molecule ($\Delta E'_{CO2*} = E_{chem} - E_{phys}$, i.e. the difference of energies between chemisorption and physisorption of second CO_2 molecule) and kinetic barriers for activating the second CO_2 molecule for the Fe_2 dimer anchored on various carbon substrates, given in the unit of eV. Related to Figure 3.

Substrate	4N-V_2	5N-V_3	6N-V_4(a)	6N-V_4(b)	6N-V_6	C_2N	g-C_3N_4
$\Delta E'_{CO2*}$	0.12	0.52	0.44	0.20	0.42	0.68	0.22
Kinetic barrier	0.29	0.54	0.83	0.87	0.67	0.70	1.04

Table S3. Zero-point energy (ZPE) and entropic correction (*TS*) at $T = 298$ K for the molecules and intermediate species involved in CO_2 reduction on Fe_2@6N-V_6. The ZPE and *TS* values were obtained from the NIST-JANAF thermodynamics table for gaseous molecules (Chase, 1998), and by calculating the vibrational frequencies for reaction intermediates (Reuter and Scheffler, 2001). The Gibbs free energy of formation for each elementary step was computed as $\Delta G = \Delta E_{DFT} + \Delta ZPE - T\Delta S$ (ΔE_{DFT} is the DFT energy difference between the initial and final states). Our test calculations show that the (ZPE – *TS*) value of a certain intermediate is very similar for the Fe_2 dimer supported by different carbon substrates, and thus the values of Fe_2@6N-V_6 were used throughout this work. Related to Figure 5.

Species	ZPE (eV)	*TS* (eV)	ZPE – *TS* (eV)	Species	ZPE (eV)	*TS* (eV)	ZPE – *TS* (eV)
H_2	0.29	0.41	−0.12	CO.COH*	0.69	0.26	0.43
H_2O	0.60	0.59	0.01	CO.HCOH*	0.99	0.23	0.76
CO	0.14	0.62	−0.48	CO–CH*	0.57	0.15	0.42
CO_2	0.31	0.67	−0.35	COH–CH*	0.91	0.15	0.76
CH_3OH	1.39	0.79	0.60	HCO–CH*	0.86	0.09	0.79
CH_4	1.20	0.58	0.62	CO–CH_2*	0.88	0.17	0.71
C_2H_4	1.36	0.71	0.65	HCO–CH_2*	1.17	0.10	1.07
C_2H_5OH	2.11	0.82	1.09	COH–CH_2*	1.22	0.15	1.06
CO_2*	0.32	0.13	0.19	HCOH–CH_2*	1.50	0.21	1.29
COOH*	0.65	0.19	0.46	CH–CH_2*	1.07	0.10	0.97
CO*	0.20	0.14	0.06	CH_2CH_2*	1.39	0.16	1.23
CO_2.CO_2*	0.59	0.29	0.30	HCOH–CH_3*	1.81	0.22	1.59
CO_2.COOH*	0.90	0.37	0.53	C_2H_5OH*	2.16	0.24	1.92
COOH.COOH*	1.23	0.30	0.93	CO.H_2COH*	1.28	0.29	0.99
CO.COOH *	0.79	0.23	0.56	CO.CH_3OH*	1.60	0.31	1.29
CO.CO*	0.39	0.11	0.28	CO.CH_2*	0.82	0.17	0.65
CO.HCO*	0.69	0.19	0.50	CO.CH_3*	1.25	0.26	0.99

Table S4. Gibbs free energy of formation (Δ*G*) for each elementary step of CO_2 reduction on the Fe_2 dimer anchored on various carbon substrates. Related to Figure 5.

Reaction	Δ*G* (eV)						
	$4N\text{-}V_2$	$5N\text{-}V_3$	$6N\text{-}V_4$ (a)	$6N\text{-}V_4$ (b)	$6N\text{-}V_6$	C_2N	$g\text{-}C_3N_4$
$2CO_2 \rightarrow CO_2.CO_2*$	−0.31	0.09	0.74	−0.18	0.48	0.67	−0.64
$CO_2.CO_2* \rightarrow CO_2.COOH*$	0.14	0.24	0.57	0.22	0.38	0.01	0.27
$CO_2.COOH* \rightarrow COOH.COOH*$	0.66	0.23	0.37	0.51	−0.80	0.39	0.87
$COOH.COOH* \rightarrow COOH.CO* + H_2O$	0.07	−0.64	−0.97	−0.80	−0.39	−1.10	−1.22
$COOH.CO* \rightarrow CO.CO* + H_2O$	−1.71	−0.90	−1.09	−0.96	−0.79	−0.95	−0.88
$CO.CO* \rightarrow CO.HCO*$	0.77	0.49	0.70	0.53	0.57	0.70	1.01
$CO.CO* \rightarrow CO.COH*$	1.34	1.10	1.83	1.13	0.88	0.76	1.03
$HCO.CO* \rightarrow CO.HCOH*$	0.57	0.64	0.82	0.38	0.31	0.03	0.32
$CO.HCOH* \rightarrow CO{-}CH* + H_2O$	−0.27	−0.49	−1.17	−0.27	−0.28	−0.30	−0.81
$CO.HCOH* \rightarrow CO.H_2COH*$	−1.10	−0.73	−0.95	−0.72	−0.27	0.94	0.31
$CO{-}CH* \rightarrow CO{-}CH_2*$	−1.08	−0.32	0.03	−0.49	−0.49	−0.15	−0.04
$CO.H_2COH* \rightarrow CO.CH_2* + H_2O$	0.44	0.41	−0.50	0.85	0.36	−1.46	−1.22
$CO.H_2COH* \rightarrow CO.CH_3OH*$	−0.35	−0.24	−0.59	−0.07	−0.79	−1.89	−1.39
$CO{-}CH_2* \rightarrow HCO{-}CH_2*$	−0.13	0.35	−0.13	−0.66	0.13	−0.52	−0.78

$CO{-}CH_2^* \rightarrow COH{-}CH_2^*$	0.28	0.14	0.32	0.28	0.28	0.46	0.10
$CO.\ CH_2^* \rightarrow CO.\ CH_3^*$	−0.94	−0.97	0.56	−1.19	−1.06	−0.21	−0.33
$HCO{-}CH_2^* \rightarrow HCOH{-}CH_2^*$	0.36	−0.18	0.15	1.06	−0.02	0.48	0.82
$COH{-}CH_2^* \rightarrow HCOH{-}CH_2^*$	−0.06	0.04	−0.30	0.12	−0.26	−0.50	−0.06
$CO.\ CH_3^* \rightarrow CO.\ CH_4^*$	−0.53	0.39	−2.04	0.65	−1.33	−1.27	−0.90
$HCOH{-}CH_2^* \rightarrow CH{-}CH_2^* + H_2O$	−0.74	−0.59	−0.23	0.22	−0.21	−0.79	−0.46
$HCOH{-}CH_2^* \rightarrow HCOH{-}CH_3^*$	0.03	0.18	−0.22	−0.03	0.01	0.02	−0.03
$CH{-}CH_2^* \rightarrow CH_2CH_2^*$	0.04	−0.26	−0.41	−1.04	0.14	0.05	−0.54
$HCOH{-}CH_2^* \rightarrow C_2H_5OH^*$	0.41	−0.55	−0.16	−0.30	0.73	−0.12	0.36
$CH_2CH_2^* \rightarrow C_2H_4$	1.25	0.96	0.23	1.11	0.58	1.11	1.69
$C_2H_5OH^* \rightarrow C_2H_5OH$	0.11	0.46	−0.04	0.59	−0.26	0.46	0.34

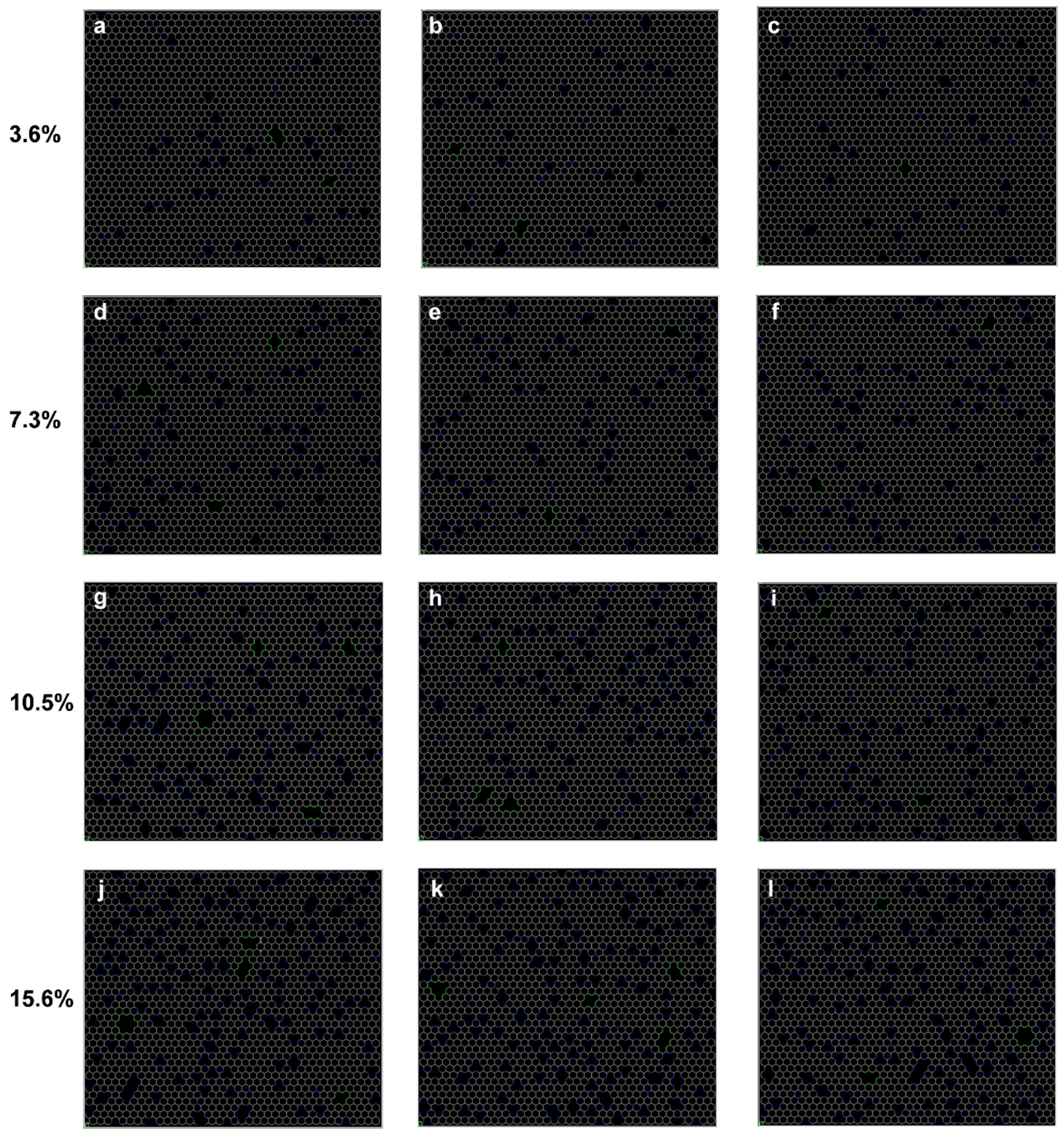

Figure S1. The N-doped carbon monolayer structures with various N content of (a, b, c) 3.6%, (d, e, f) 7.3%, (g, h, i) 10.5% and (j, k, l) 15.6%, respectively, created by our home-made program that randomly introduces pyridinic N and graphitic N into graphene lattice. The results show that 4N-V_2, 5N-V_3 and 6N-V_4 exist as the N content lower than 7.5% and 6N-V_6 configuration emerges as the N doping content reaches 10%. The C and N atoms are shown in grey and blue/green colors, respectively. The green highlights the structural models considered in this work. Related to Figure 1.

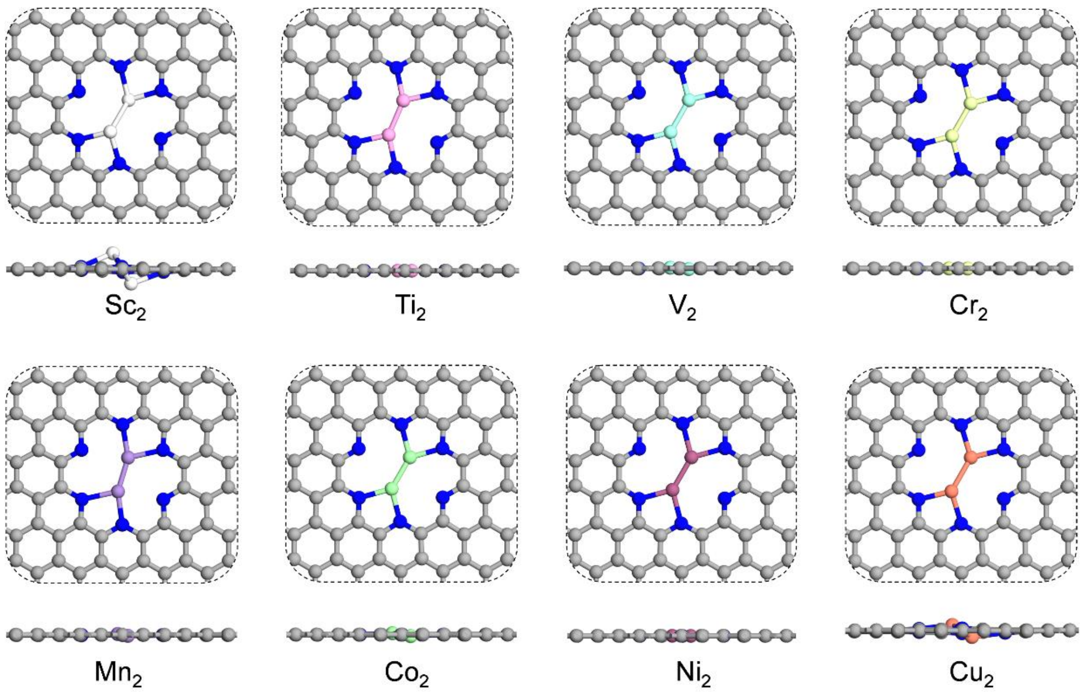

Figure S2. Atomic structures of 3*d* transition metal dimers anchored on the 6N-V_6 monolayer. The C and N atoms are shown in grey and blue colors, respectively. Related to Table 2.

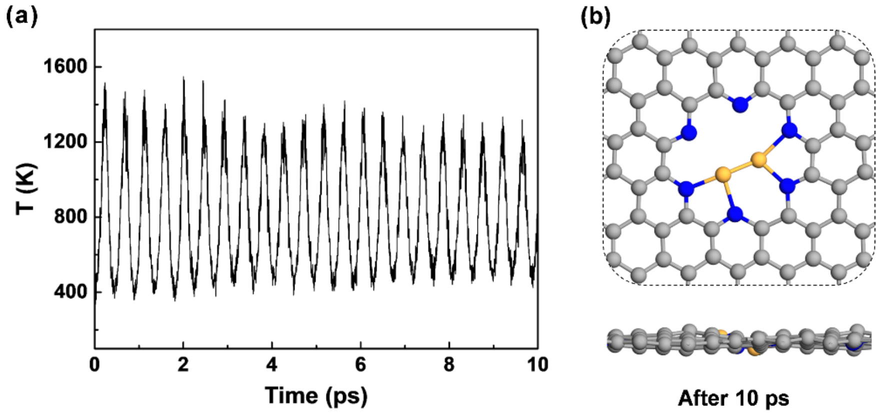

Figure S3. (a) Temperature profile and (b) snapshot atomic structure of Fe_2@6N-V_6 from *ab initio* molecular dynamics simulation at 800 K for 10 ps, which well maintains the initial equilibrium geometry with an average atomic displacement of 0.16 Å in the vertical direction. The C, N and Fe atoms are shown in grey, blue and orange colors, respectively. Related to Figure 1.

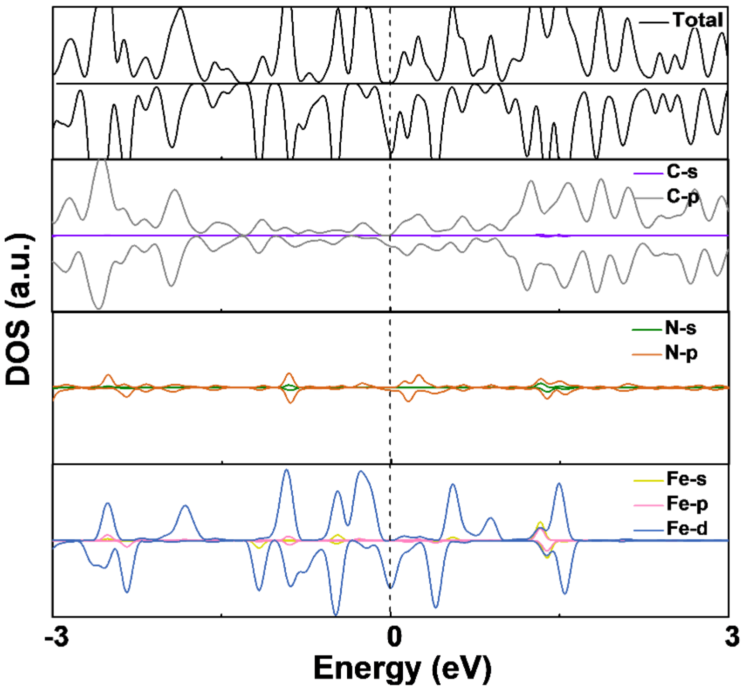

Figure S4. The projected density of states (DOS) of Fe_2@6N-V_6. The dashed line shows the Fermi level. Related to Figure 2.

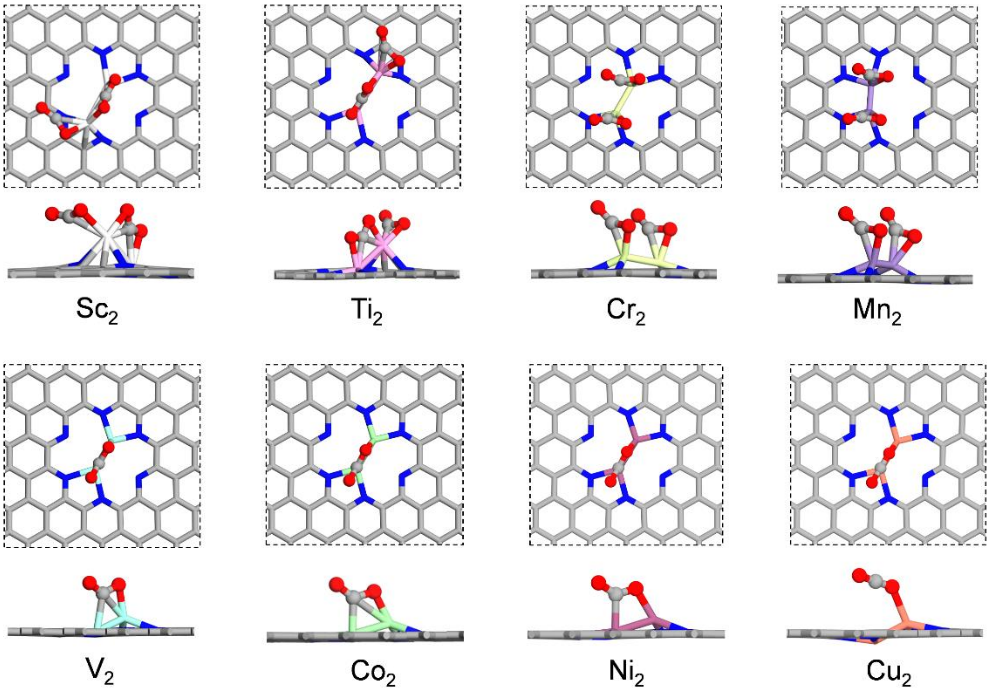

Figure S5. Atomic structures of dual or single CO_2 molecule adsorbed on 3*d* transition metal dimers anchored on the 6N-V_6 monolayer. The C, N and O atoms are shown in grey, blue and red colors, respectively. Related to Table 2.

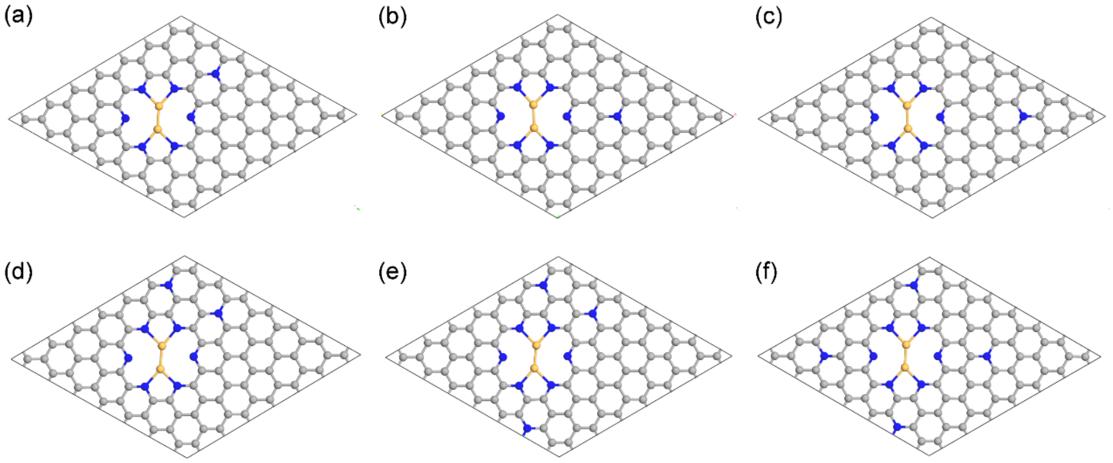

Figure S6. Model structures of Fe_2@6N-V_6 with presence of the graphitic N atoms near or away from the hole. For models (a), (b) and (c), we placed the graphitic N atom at different distances from the Fe_2 dimer, with $d_a < d_b < d_c$. For models (d), (e) and (f), we considered two, three and four graphitic N atoms per supercell, respectively. The C, N and Fe atoms are shown in grey, blue and orange colors, respectively. Related to Figure 1.

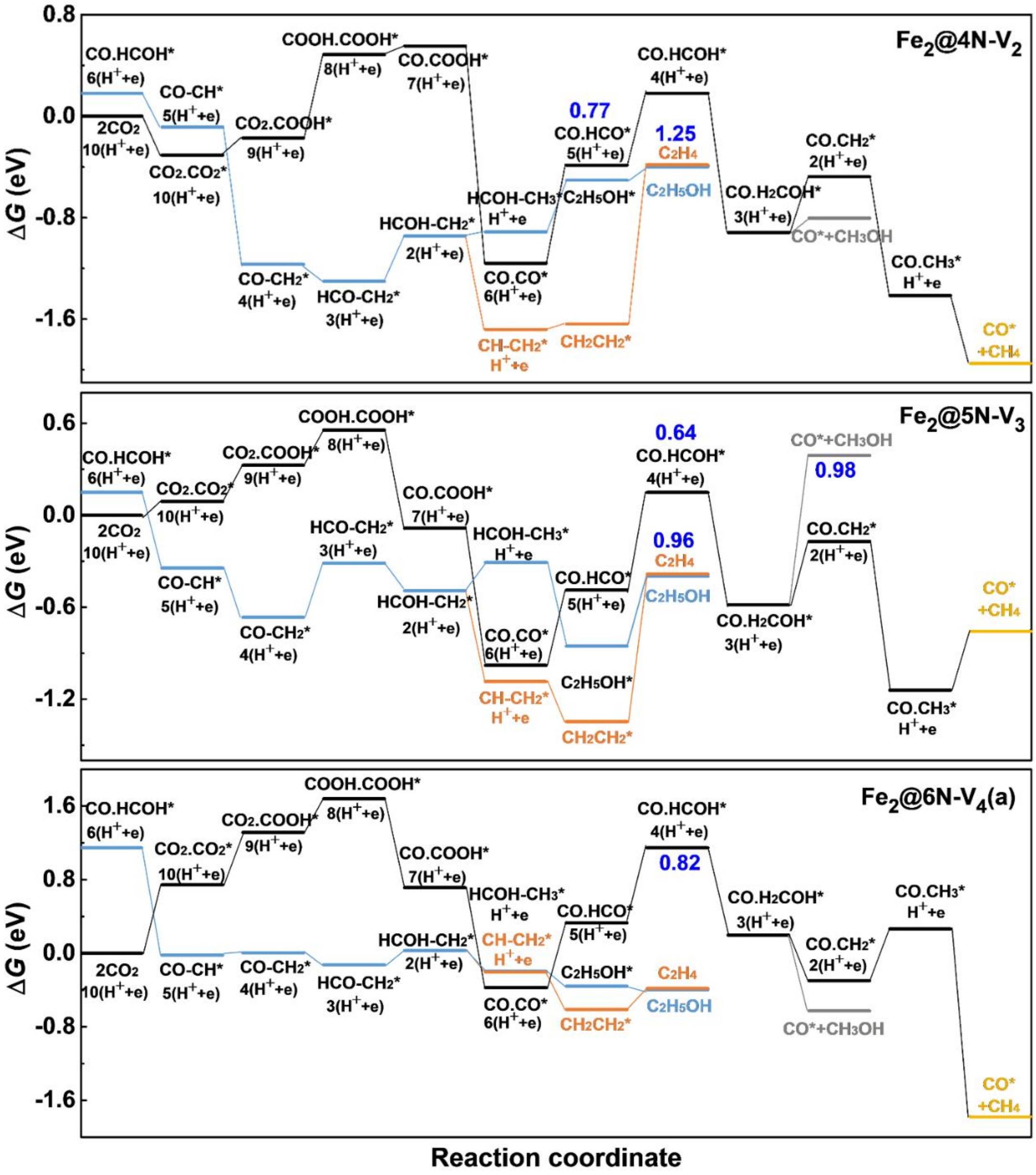

Figure S7. Free energy diagrams of CO_2 reduction to form various C_1 and C_2 products (indicated by different colors) on Fe_2@4N-V_2, Fe_2@5N-V_3 and Fe_2@6N-V_4(a). The blue numbers indicate ΔG_{RDS} values for every products. Related to Figure 5.

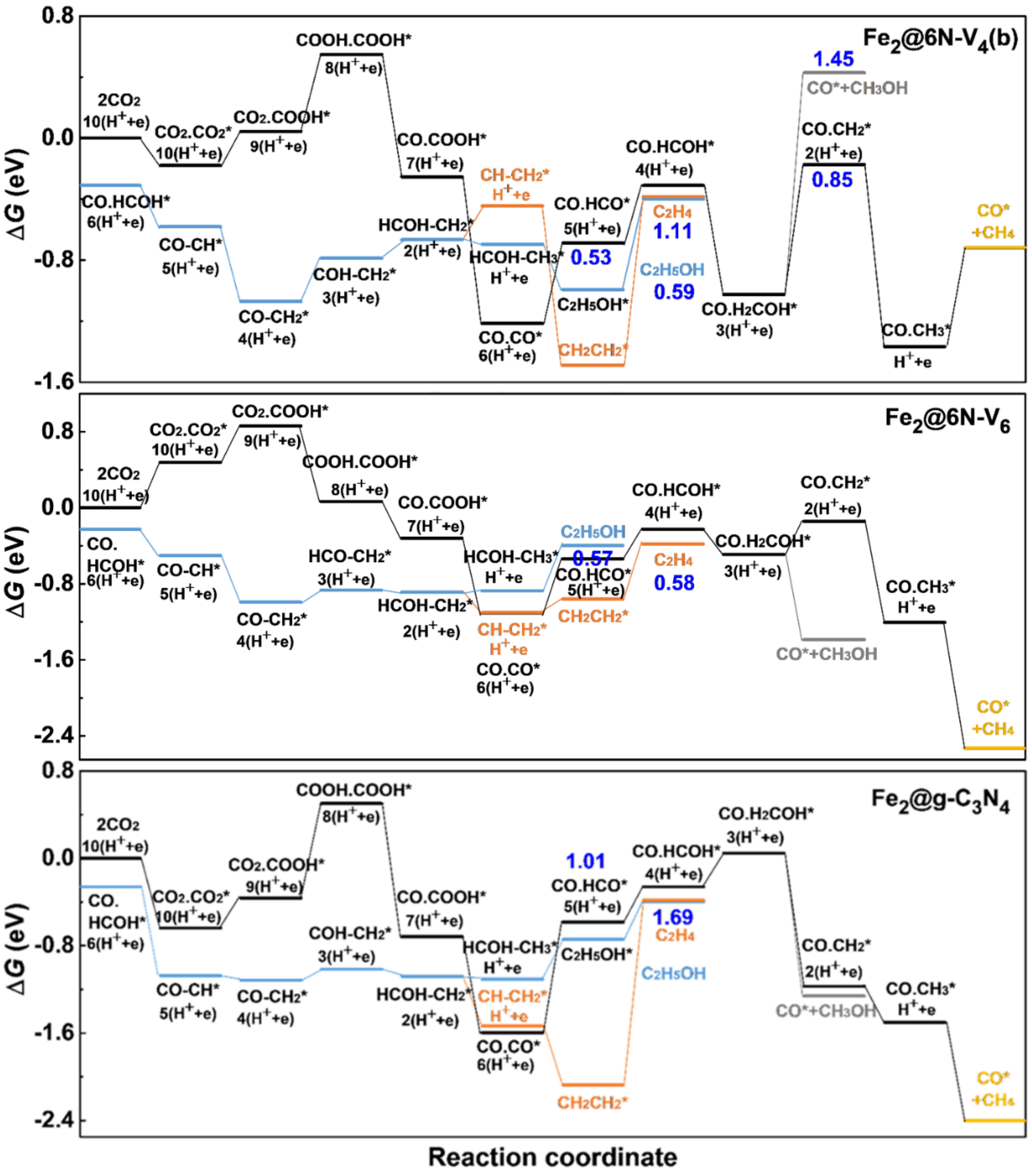

Figure S8. Free energy diagram of CO_2 reduction to form various C_1 and C_2 products (indicated by different colors) on Fe_2@6N-V_4(b), Fe_2@6N-V_6 and Fe_2@g-C_3N_4. The blue numbers indicate ΔG_{RDS} values for every products. Related to Figure 5.

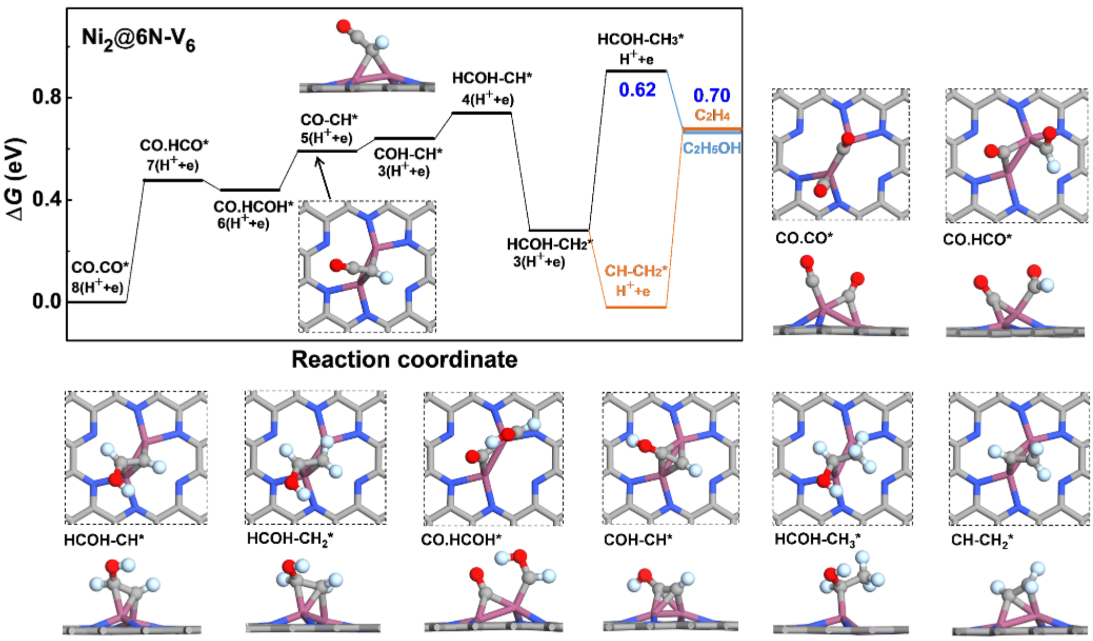

Figure S9. Free energy diagram and atomic structures of CO_2 reduction to form C_2H_4 and C_2H_5OH products (indicated by different colors) on Ni_2@6N-V_6, the free energies of rate-determining step are 0.70 and 0.62 eV for C_2H_4 and C_2H_5OH products, respectively. The blue numbers indicate ΔG_{RDS} values for every products. The H, C, N, O and Ni atoms are shown in light blue, grey, blue, red and claret colors, respectively. Related to Figure 5.

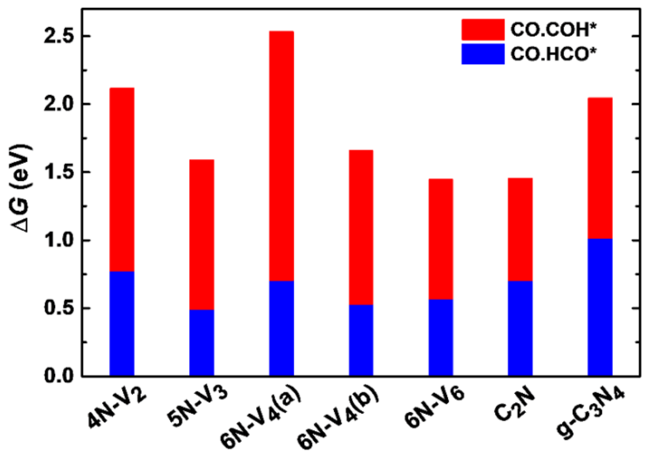

Figure S10. Gibbs free energy of formation (ΔG) for generating CO.HCO* and CO.COH* intermediates from protonation of CO.CO*. Related to Figure 4.

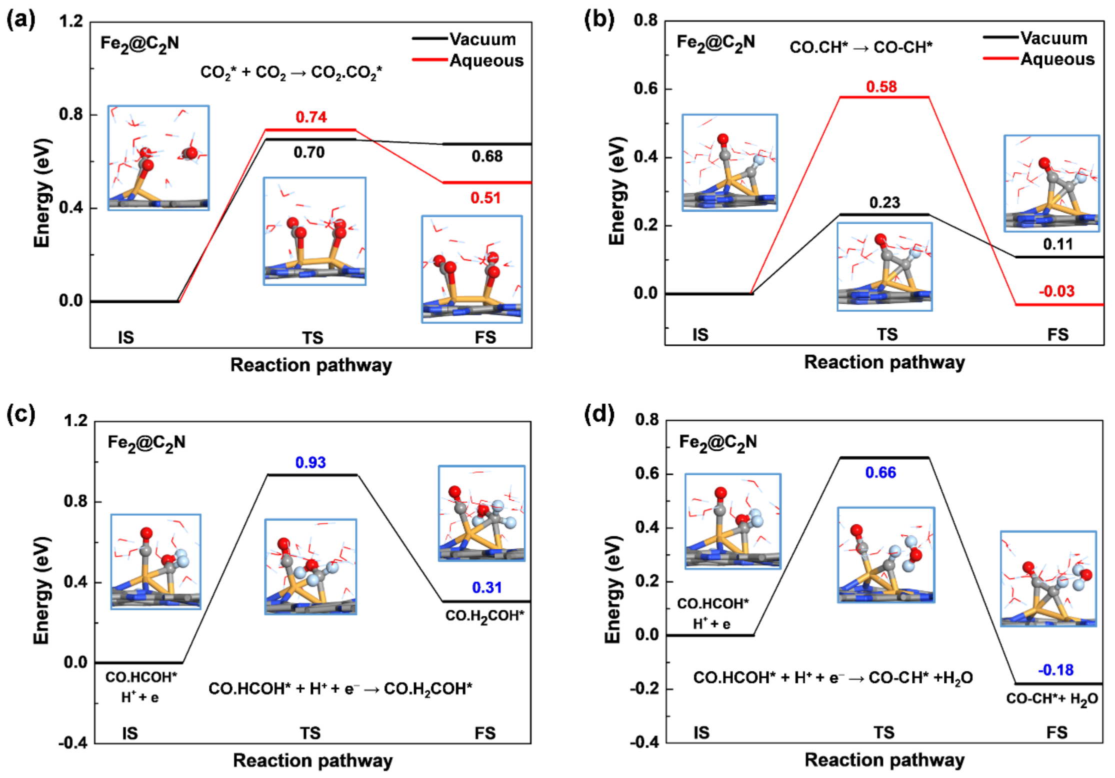

Figure S11. (a) The kinetic process of second CO_2 activation in vacuum and in the aqueous condition. (b) The CO−CH* coupling in vacuum and in the aqueous condition. Competing reactions of CO.HCOH* to form (c) C_1 and (d) C_2 intermediates on Fe_2@C_2N in water environment, respectively. The insets display the structures of initial state (IS), transition state (TS) and final state (FS). The numbers give the kinetic barriers (middle) and heat of reaction (right). The H, C, N, O and Fe atoms are shown in light blue, grey, blue, red and orange colors, respectively. Related to Table 3.

References

Kresse, G., Furthmüller, J. (1996). Efficient Iterative Schemes for *ab initio* Total-Energy Calculations Using a Plane-Wave Basis Set. Phys. Rev. B 54, 11169-11186.

Kresse, G., Joubert, D. (1999). From Utrasoft Pseudopotentials to the Projector Augmented-Wave Method. Phys. Rev. B 59, 1758-1775.

Perdew J. P., Burke, K., Ernzerhof M. (1996). Generalized Gradient Approximation Made Simple. Phys. Rev. Lett. 77, 3865-3868.

Grimme, S., Antony, J., Ehrlich, S., Krieg, H. (2010). A Consistent and Accurate *ab initio* Parametrization of Density Functional Dispersion Correction (DFT-D) for the 94 Elements H-Pu. J. Chem. Phys. 132, 154104.

Mulliken, R. S. (1955). Electronic Population Analysis on LCAO–MO Molecular Wave Functions. II. Overlap Populations, Bond Orders, and Covalent Bond Energies. J. Chem. Phys. 23, 1841-1846.

Clark, S. J., Segall, M. D., Pickard, C. J., Hasnip, P. J., Probert, M. I., Refson, K., Payne, M. C. (2005). First Principles Methods Using CASTEP. Z. Kristallogr. –Cryst. Mater. 220, 567-570.

Henkelman, G., Uberuaga, B. P., Jónsson, H. (2000). A climbing image nudged elastic band method for finding saddle points and minimum energy paths. J. Chem. Phys. 113, 9901-9904.

Peterson, A. A., Abild-Pedersen, F., Studt, F., Rossmeisl, J., Nørskov, J. K. (2010). How Copper Catalyzes the Electroreduction of Carbon Dioxide into Hydrocarbon Fuels. Energy Environ. Sci. 3, 1311-1315.

Sheng, Z. H., Shao, L., Chen, J. J., Bao, W. J., Wang, F. B., Xia, X. H. (2011). Catalyst-Free Synthesis of Nitrogen-Doped Graphene Via Thermal Annealing Graphite Oxide with Melamine and Its Excellent Electrocatalysis. ACS Nano 5, 4350-4358.

Sarau, G., Heilmann, M., Bashouti, M., Latzel, M., Tessarek, C., Christiansen, S. (2017). Efficient Nitrogen Doping of Single-Layer Graphene Accompanied by Negligible Defect Generation for Integration into Hybrid Semiconductor Heterostructures. ACS Appl. Mater. Interfaces 9, 10003-10011.

Chase, M. W. (1998). NIST-JANAF Thermochemical Tables. American Chemical Society, New York.

Reuter, K.; Scheffler, M. (2001). Composition, Structure, and Stability of RuO_2 (110) as a Function of Oxygen Pressure. Phys. Rev. B 65, 035406.
